# Dietary 5-hydroxytryptophan improves sheep growth performance by enhancing ruminal functions, antioxidant capacity, and tryptophan metabolism: *in vitro* and *in vivo* studies

**DOI:** 10.3389/fimmu.2024.1398310

**Published:** 2024-05-21

**Authors:** Zhe Sun, Natnael D. Aschalew, Long Cheng, Yuanhong Xia, Longyu Zhang, Guopei Yin, Shikun Wang, Ziyuan Wang, Jianan Dong, Weigang Zhang, Wei Zhao, Guixin Qin, Xuefeng Zhang, Rongzhen Zhong, Tao Wang, Yuguo Zhen

**Affiliations:** ^1^ Jilin Agricultural University (JLAU)-Borui Dairy Science and Technology R&D Center, Key Laboratory of Animal Nutrition and Feed Science of Jilin Province, College of Animal Science and Technology, Jilin Agricultural University, Changchun, China; ^2^ Key Laboratory of Animal Production Product Quality and Security Ministry of Education, College of Animal Science and Technology, Jilin Agricultural University, Changchun, China; ^3^ College of Life Sciences, Engineering Research Center of Bioreactor and Pharmaceutical Development, Ministry of Education, Jilin Agricultural University, Changchun, China; ^4^ Postdoctoral Scientific Research Workstation, Feed Engineering Technology Research Center of Jilin Province, Changchun Borui Science & Technology Co. Ltd., Changchun, China; ^5^ College of Agriculture and Environmental Science, Dilla University, Dilla, Ethiopia; ^6^ Jilin Province Feed Processing and Ruminant Precision Breeding Cross-Regional Cooperation Technology Innovation Center, Jilin Provincial Key Laboratory of Grassland Farming, Northeast Institute of Geography and Agroecology, Chinese Academy of Sciences, Changchun, China

**Keywords:** 5-hydroxytryptophan, sheep, ruminal fermentation, microbiota, growth performance, metabolites

## Abstract

**Background:**

Hydroxytryptophan (5-HTP) can regulate the synthesis of 5-Hydroxytryptamine (5-HT) and melatonin (MT). In a previous metabolome analysis, we found that 5-HTP is an effective ingredient in yeast culture for regulating rumen fermentation. However, research on the effect of this microbial product (5-HTP) as a functional feed additive in sheep production is still not well explained. Therefore, this study examined the effects of 5-HTP on sheep rumen function and growth performance using *in vitro* and *in vivo* models.

**Methods:**

A two-factor in vitro experiment involving different 5-HTP doses and fermentation times was conducted. Then, in the *in vivo* experiment, 10 sheep were divided into a control group which was fed a basal diet, and a 5-HTP group supplemented with 8 mg/kg 5-HTP for 60 days.

**Results:**

The results showed that 5-HTP supplementation had a significant effect on *in vitro* DMD, pH, NH_3_-N, acetic acid, propionic acid, and TVFA concentrations. 5-HTP altered rumen bacteria composition and diversity indices including Chao1, Shannon, and Simpson. Moreover, the *in vivo* study on sheep confirmed that supplementing with 8 mg/kg of 5-HTP improved rumen fermentation efficiency and microbial composition. This led to enhanced sheep growth performance and increased involvement in the tryptophan metabolic pathway, suggesting potential benefits.

**Conclusion:**

Dietary 5-HTP (8 mg/kg DM) improves sheep growth performance by enhancing ruminal functions, antioxidant capacity, and tryptophan metabolism. This study can provide a foundation for the development of 5-HTP as a functional feed additive in ruminants’ production.

## 1 Introduction

5-Hydroxytryptophan (5-HTP) can regulate the synthesis of 5-Hydroxytryptamine (5-HT) and melatonin (MT). 5-HT and its derivatives have been recognized as potential indicators of Alzheimer’s disease progression along the microbiota-gut-brain axis (1). Melatonin, a popular, commercially available drug, is derived from 5-HTP (2) and regulates 5-HT levels in the body. Decreased 5-HT levels can cause insomnia. 5-HTP regulates 5-HT production, improves sleep in animals (3), and relieves depression (4). 5-HTP has antioxidant, anti-inflammatory, and analgesic properties with higher hydroxyl radical-scavenging effects than vitamin C (5). It maintains membrane fluidity during oxidative stress (6) and alleviates hyperglycaemia-induced oxidative stress (7). Tryptophan (Trp), an essential amino acid, is crucial for animal production and the maintenance of immune function. Its metabolite, 5-HT, regulates the microbial composition of the digestive tract and helps maintain the immune function of the body (8). Trp can affect animal feed intake and ingestion as a raw material for tissue protein synthesis (9). Trp supplementation in the rumen of dairy cows can lead to an increased milk yield, higher levels of blood growth hormones, improved antioxidant capacity, and increased MT content (10). Trp can effectively reduce urinary nitrogen excretion and increase nitrogen retention in the meat of growing lambs (11). 5-HTP suppresses the release of gastric hydrochloric acid (12), increases gastrointestinal motility (13), and is rapidly absorbed by most tissues (14). Zhao et al. (15) found that rumen-protected 5-HTP supplementation increased blood 5-HTP and MT content in sheep during the day and night, respectively, and increased MT synthesis in the pineal gland and intestine. 5-HTP perfusion in the intestinal tract and blood of sheep significantly increases MT levels, antioxidant capacity in the mucosa, liver, muscle, adipose tissue, abdominal and tail fat, and MT content in the liver and muscle (16). Prenatal injection of 5-HTP reduced the casein content in dairy goat colostrum on the day of parturition, suggesting that 5-HT can control milk protein synthesis during the perinatal period (17). 5-HTP supplementation enhances milk yield, plasma growth hormone, prolactin, and insulin content in lactating dairy cows (18), whereas intragastric infusion increases serum 5-HT content in Holstein steers (19). In a previous metabolome analysis, we found that 5-HTP is an effective ingredient in yeast culture for regulating rumen fermentation (20). However, research on the effect of this microbial products (5-HTP) as a functional feed additive in sheep production has not yet been well explained. Therefore, in this study, the interactions between 5-HTP and sheep was elucidated based on *in vitro* and *in vivo* models.

## 2 Material and methods

### 2.1 Animal ethics statement

All experimental procedures followed the Guidelines for the Care and Use of Experimental Animals at Jilin Agricultural University (JLAU-ACUC2022-003, Changchun, China).

### 2.2 Experimental design and animals

5-HTP was purchased from Shanghai Macklin Biochemical Co., Ltd. with a purity of 99% and stored at 2-8°C. Before the start of the experiment, the diets used for *in vitro* and *in vivo* experiments was tested for Trp and 5-HTP content by Qingdao Kechuang Quality Inspection Company. The results showed that the Trp content was 92.77 μg/g, and the 5-HTP content was not detectable (<0.6 μg/g).

In the *in vitro* study, a two-factor experimental design was applied, with 5-HTP doses (0, 2, 4, 8, and 10 mg/kg DM) and fermentation times (0, 3, 6, 12, 24, and 48 h) as factors. Three rumen-cannulated short-tailed Han sheep rams with an average body weight of 30 ± 2.5 kg, were utilized as rumen fluid donors.

The *in vitro* results showed that 5-HTP supplementation had a significant effect on *in vitro* DMD, ruminal pH, NH3-N, acetic acid, propionic acid, and TVFA concentrations. Therefore, 8 mg/kg 5-HTP was chosen in the *in vivo* experiments. A total of ten short-tailed Han sheep rams with an average body weight of 20 ± 1.5 kg were used in a univariate experimental design. The sheep were divided into the control and 5-HTP groups, with five sheep in each group. The control group received a basal diet only (**Supplementary Table S1**), and the 5-HTP group was supplemented with 8 mg/kg DM 5-HTP. The sheep were housed in individual pens with wire partitions and bamboo flooring raised 40 cm above the ground. The sheep were given 15 days to adapt to the basal diet before the beginning of the 60-day experimental period.

### 2.3 Rumen fermentation parameters *in vitro*

Rumen fluid was collected before morning feeding using a rigid PVC tube from different points in the rumen. The fluid was then transferred to a warmed (39°C) thermos flask that had been purged with CO_2_ for 10 min. The fluid was filtered through a four-layer cheesecloth. An ANKOM^RF^ Gas Production System was used to measure gas production during fermentation. Each sample (2.00 g) was placed in an 800-mesh nylon bag and heat-sealed. The bag was placed in a 250-mL ANKOM bottle and 120 mL of buffer-rumen solution was added according to the method described by Menke (21). The bottles were incubated in a shaker at 39°C and 80 rpm for fermentation. Samples were collected at 0, 3, 6, 12, 24, and 48 h for *in vitro* digestion and rumen fermentation analysis, and at 0, 12, and 48 h for microbial composition and metabolite analysis. There were 12 replicates for each sampling time. The pH level was measured using a SANXIN MP523-04 portable pH meter (Shanghai Sanxin Instrument Co., Ltd., Shanghai, China). Ammonia nitrogen (NH_3_-N) concentration was determined using a colorimetric method (22). A Shimadzu UV-1201 UV colorimetric Spectrophotometer (Shimadzu, Tokyo, Japan) was used for colorimetry. Volatile fatty acids (VFA) were measured using an Agilent 7890 B gas chromatography system (Agilent Technologies, Santa Clara, CA, USA) (23). Gas production was performed using an ANKOM^RF^ Gas Production System automatically recorded by GPM software. The amount of gas production was calculated according to the formula:

Vy=Vx× Ppsi×0.068004084 , where: Vy = 39°C gas production volume, mL; Vx = the gas volume in the space above the culture medium in each sample bottle, mL; Ppsi = GPM software automatically recorded 24 h cumulative pressure, psi. To determine the dry matter degradation (DMD) rate, the nylon filter bag was washed with cold water, air-dried for 24 h, and then oven-dried at 65°C until a constant weight was achieved. Thereafter, the DMD rate was determined using the formula:


$$\text{DMD rate}(\%)=\frac{A-\left(B-W\times K\right)}{A}\times 100$$

A = substrate weight before fermentation (dry matter basis)

B = sample + nylon bag weight after fermentation (dry matter basis)

W = nylon bag weight after fermentation (after drying at 105 °C)

K = nylon bag factor (weight after drying at 105 °C after fermentation/weight before fermentation)

### 2.4 Microbial DNA extraction, PCR amplification, sequencing, and analysis *in vitro*

Rumen fluid samples for high-throughput sequencing were sent to Shanghai Personal Biotechnology Co., Ltd. (Shanghai, China). Nucleic acids were extracted using the OMEGA Soil DNA Kit and DNA was quantified using a Nanodrop. The V3-V4 region of the bacterial 16S rRNA gene was selected for sequencing. PCR amplification of the bacterial 16S rDNA V3-V4 specific primer sequences, 338F (5′-ACTCCTACGGGAGGCAGCA-3′) and 806R (5′-GGACTACHVGGGTWTCTAAT-3′) was performed using the NEB Q5 DNA high-fidelity polymerase.

Sequencing libraries were prepared using the Illumina TruSeq Nano DNA LT preparation kit designed for high-throughput sequencing. Sequencing data were primarily analyzed using the DADA2 method after the machine was exited for steps such as primer removal, quality filtering, denoising, splicing, and chimerism removal (24). Alpha diversity indices were calculated using QIIME2 (2019.4) and R language using the ggplot2 package. Beta-diversity analysis was conducted by calculating the differences between samples. The abundance values of metabolic pathways were determined using the Kyoto Encyclopedia of Genes and Genomes (KEGG) database, MetaCyc database, and three commonly used metabolic pathway databases in COG data, followed by metabolic pathway difference analysis.

### 2.5 Rumen metabolite analysis *in vitro*

#### 2.5.1 Sample pretreatment

A sample (200 μL) was collected to which 800 μL of precooled extraction solution (methanol and ribitol) was added, and vortexed for 30 seconds. The samples were sonicated in an ice-water bath for 10 min. After centrifugation at 12,000 rpm and 4°C for 15 min, 400 μL of the supernatant was transferred into a 1.5 mL Eppendorf tube. The quality control (QC) samples were combined with a 70 μL sample. The extract was dried, and the dried metabolite was mixed with 40 μL of methoxyamine reagent. Each sample received 50 μL of bis-(trimethylsilyl)trifluoroacetamide, and the mixture was incubated for 1.5 h at 70°C. Fatty acid methyl esters (FAMEs) were added to the mixed samples and tested randomly.

#### 2.5.2 GC-TOF-MS analysis

The Agilent 7890 gas chromatography-time-of-flight mass spectrometer was equipped with an Agilent DB-5MS capillary column (30 m × 250 μm × 0.25 μm, JampW Scientific, Folsom, CA, USA), GC-TOF-MS was applied (25).

### 2.6 Growth performance of sheep

The dry matter intake (DMI, kg/day) and body weights (kg) were measured before the morning feeding on days 0, 15, 30, 45, and 60. The average daily gain (kg/d) and total body weight gain (kg) were calculated accordingly.

### 2.7 Blood biochemical indices in sheep

Serum samples were collected from the jugular vein on days 0, 15, 30, 45, and 60, and then centrifuged at 3000 rpm and 4°C for 10 min. The supernatant was stored at -20°C until analysis. Blood biochemical indices were analyzed at the Beijing Huaying Institute of Biotechnology (Beijing, China). Blood antioxidant indicators, including total antioxidant capacity (T-AOC, HY-60021 kit), superoxide dismutase (SOD, HY-60001 kit), glutathione peroxidase (GSH-PX, HY-60005 kit), and catalase (CAT, HY-M0018 kit), and malondialdehyde (MDA, HY-60003 kit). Blood hormones, such as insulin (INS, HY-D0001 kit) and growth hormones (GH, HY-C0018 kit). The levels of 5-HT (HY-157 kit), 5-HTP (HY-10890 kit), and MT (HY-D0040 kit).

### 2.8 Blood metabolite analysis in sheep

#### 2.8.1 Sample pretreatment

The sample (100 μL) was transferred into an Eppendorf tube with a 400 μL mixture of methanol and acetonitrile (1:1, V/V). Finally, the mixture was vortexed for 30 s. The sample was sonicated for 10 min while being immersed in an ice water bath and maintained at -40°C for an hour. Subsequently, the sample was subjected to centrifugation at 4°C and 12,000 rpm for 15 min. The supernatant was transferred to an injection bottle for testing. The supernatant was combined with quality control samples and analyzed together.

#### 2.8.2 LC-MS analytical conditions

The Vanquish ultra-high-performance liquid chromatograph (Thermo Fisher Scientific) is equipped with a Waters ACQUITY UPLC BEH Amide (2.1 mm × 100 mm, 1.7 μm) liquid chromatographic column for separating the target compounds (26). Phase A of the LC was aqueous and contained 25 mM ammonium acetate and 25 mM ammonia, whereas phase B contained acetonitrile. The sample disc temperature was 4°C, and the injection volume was 2 μL. A Thermo Q Exactive HFX mass spectrometer was used to collect primary and secondary mass spectral data. The parameters included a sheath gas flow rate of 30 Arb, an auxiliary gas flow rate of 25 Arb, a capillary temperature of 350°C, a full MS resolution of 120,000, an MS/MS resolution of 7,500, collision energy of 10/30/60 in NCE mode, and a spray voltage of 3.6 kV (positive) or -3.2 kV (negative).

### 2.9 Statistical analysis

Statistical analysis for all repeated measurements was performed using SPSS software version 27 General Linear Model. The *in vitro* variables were examined using two-way ANOVA and significance levels were evaluated using Duncan’s multiple-range test. The *in vivo* data were analyzed using the independent sample t-test and presented as mean ± SEM. A significant difference was defined as *P* < 0.05, and a highly significant difference was defined as *P* < 0.01. Additionally, we used the ChromaTOF software, ProteoWizard software, and a self-written R program package (kernel: XCMS) (26).

## 3 Results

### 3.1 Rumen fermentation parameters *in vitro*

The pH, ammonia nitrogen (NH_3_-N), DMD, and gas production results are presented in **Table 1**. The pH of the 5-HTP group decreased significantly during the fermentation period (*P* < 0.01). The NH_3_-N concentration was influenced by both 5-HTP dose (*P* < 0.05) and fermentation time (*P* < 0.01). At 12 h, the DMD rates were higher in the high-dose groups (8 mg/kg and 10 mg/kg). Additionally, in the 8 mg/kg group, DMD was greater at 24 and 48 h compared to the 10 mg/kg group (*P* < 0.05). The 5-HTP supplement had a significant effect on gas production (*P* < 0.05), with higher levels observed in the 8 mg/kg group at 12, 24, and 48 h. Furthermore, it also significantly affected acetic acid (*P* < 0.05) and total volatile fatty acid (TVFA) production (*P* < 0.01, **Table 2**). The TVFA remained higher in the 8 mg/kg group at 12, 24, and 48 h, whereas it was lower in the 4 mg/kg group at 24 and 48 h, indicating a significant interaction effect (*P* < 0.01).

**Table 1:** Effects of 5-HTP supplementation on rumen pH, NH_3_-N concentration (mg/dL), DMD (%, DM), and gas production (mL) *in vitro*.

Item	Time	5-HTP group (mg/kg DM)					SEM	*P*-value		
		0	2	4	8	10		Dose	Time	D×T
pH value	3h	6.63	6.63	6.63	6.61	6.65	0.01	0.865	<0.01	0.844
	6h	6.54	6.51	6.51	6.51	6.57	0.01			
	12h	6.37	6.39	6.38	6.40	6.31	0.02			
	24h	6.22	6.18	6.20	6.20	6.22	0.02			
	48h	6.01	6.02	6.02	6.02	6.10	0.02			
NH_3_-N	3h	18.95^ab^	22.04^a^	22.00^a^	16.27^b^	14.11^b^	0.89	< 0.05	<0.01	<0.01
	6h	24.40^a^	24.68^a^	25.73^a^	16.91^b^	14.70^b^	1.08			
	12h	36.06^a^	33.95^a^	37.73^a^	34.58^a^	21.13^b^	1.46			
	24h	55.84^ab^	57.79^a^	48.93^b^	55.83^ab^	57.46^a^	1.22			
	48h	67.42	65.06	64.91	69.04	71.67	1.23			
DMD	3h	17.76	17.25	17.91	18.47	18.71	0.62	0.280	<0.01	0.353
	6h	23.13^ab^	25.85^a^	23.25^ab^	23.36^ab^	20.78^b^	0.60			
	12h	31.23	30.71	30.54	30.84	31.45	0.61			
	24h	37.53^ab^	38.90^ab^	37.65^ab^	40.90^a^	36.50^b^	0.57			
	48h	49.70^a^	46.63^ab^	49.00^ab^	47.36^ab^	44.71^b^	0.65			
Gas production	3h	49.14	50.99	53.80	51.99	50.87	1.92	<0.05	<0.01	0.051
	6h	95.10^ab^	90.89^ab^	106.73^a^	81.96^b^	87.33^ab^	3.53			
	12h	155.69	159.31	151.40	166.33	153.44	5.22			
	24h	226.22^ab^	234.82^ab^	193.68^b^	252.46^a^	196.23^b^	6.94			
	48h	291.52	288.75	280.39	317.11	276.59	7.06			

^a,b^Different superscripts in the same raw implies their mean values are significantly different (P ≤ 0.05). Where: D×T- dose and fermentation time interaction; DMD - dry matter degradation rate.

**Table 2:** Effects of 5-HTP supplementation on volatile fatty acids concentration (mmol/L) *in vitro*.

Item	Time	5-HTP group (mg/kg DM)					SEM	*P*-value		
		0	2	4	8	10		Dose	Time	D×T
Acetic acid	3h	19.32	19.91	19.34	21.16	20.35	0.77	<0.05	<0.01	0.731
	6h	24.84	27.61	25.54	25.77	25.17	0.83			
	12h	32.41	32.34	32.60	39.13	35.65	1.06			
	24h	38.21^b^	42.59^ab^	38.61^b^	48.93^a^	46.73^ab^	1.38			
	48h	57.50	56.18	52.09	58.24	57.28	1.47			
Propionic acid	3h	8.83	9.53	10.71	9.73	8.38	0.40	0.235	<0.01	0.692
	6h	12.06	13.85	13.93	12.19	11.77	0.53			
	12h	18.94	19.94	18.29	21.78	18.11	0.82			
	24h	25.21	25.21	23.94	30.45	28.59	0.96			
	48h	33.08	31.70	30.38	35.03	33.98	1.07			
Butyric acid	3h	4.84	4.95	5.12	5.83	6.37	0.30	0.462	<0.01	0.915
	6h	6.37	6.78	7.75	7.03	7.82	0.36			
	12h	10.09	10.25	8.93	12.37	11.01	0.57			
	24h	12.63	12.83	13.85	15.92	13.20	0.73			
	48h	17.08	18.35	21.00	17.61	17.97	0.98			
TVFA	3h	35.72	37.23	40.42	39.99	39.99	1.31	<0.01	<0.01	<0.01
	6h	46.97	51.06	59.70	49.67	50.45	1.98			
	12h	67.16	66.90	65.98	75.97	68.19	2.07			
	24h	84.80^b^	85.32^b^	70.76^c^	100.45^a^	99.80^a^	2.36			
	48h	121.37^a^	117.91^a^	99.64^b^	128.36^a^	123.21^a^	2.99			
A/P	3h	2.46	2.25	2.11	2.37	2.78	0.15	0.137	<0.01	0.962
	6h	2.26	2.09	1.95	2.40	2.77	0.14			
	12h	1.93	1.71	1.82	2.26	2.11	0.11			
	24h	1.57	1.72	1.58	1.74	1.75	0.06			
	48h	1.81	1.71	1.71	1.77	1.83	0.05			

^a,b^Different superscripts in the same raw implies their mean values are significantly different (P ≤ 0.05).Where: D×T- dose and fermentation time interaction; TVFA- Total volatile fatty acids; A/P- acetic to propionic acid ratio.

### 3.2 Rumen microbial community composition *in vitro*

**Figure 1:**
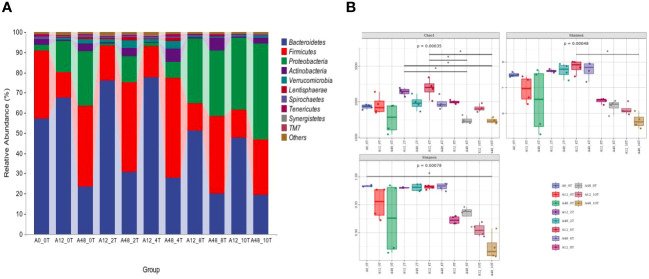
Ruminal bacteria phyla abundance **(A)** and alpha diversity indices **(B)** under 5-HTP supplementation at different fermentation times *in vitro*. * indicates a significant difference in mean between groups (*P* < 0.05).

An average of 40,591 high-quality sequences per sample was obtained from the 1,786,014 valid sequences. 5-HTP significantly altered the relative abundances of Bacteroidetes, Firmicutes, Proteobacteria, Actinobacteria, and Verrucomicrobia (*P* < 0.01; **Supplementary Table S2**). This interaction had a greater impact on the abundances of Bacteroidetes, Firmicutes (*P* < 0.01), and Verrucomicrobia (*P* < 0.05). Proteobacteria in the high-dose groups (8 mg/kg and 10 mg/kg) were more abundant at 12 and 48 h (*P* < 0.05, **Figure 1A**). *Prevotella, Succinivibrio, Selenomonas*, and *Succiniclasticum* were the predominant genera (**Supplementary Table S2**). 5-HTP significantly affected the relative abundance of the *Butyrivibrio* genus (*P* < 0.05). The interaction effects also had a significant impact on the relative abundances of *Succiniclasticum* and *Sharpea* (*P* < 0.01). Additionally, the low-dose 5-HTP groups (2 mg/kg and 4 mg/kg) were able to decrease the abundance of *Vibrio succinogenes*, whereas the high-dose 5-HTP groups (8 mg/kg and 10 mg/kg) were able to increase their abundance.

### 3.3 Rumen bacteria alpha diversity *in vitro*

Different doses of 5-HTP significantly affected the Chao1, Shannon, and Simpson indices at various fermentation times (*P* < 0.05, **Figure 1B**).

### 3.4 Beta diversity of rumen microbial communities *in vitro*

**Figure 2:**
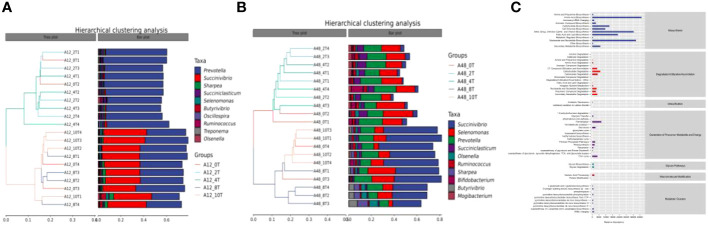
Hierarchical clustering analysis of bacterial genera at 12 **(A)** and 48 h **(B)**, metabolic pathway statistics **(C)** under 5-HTP supplementation at different fermentation times *in vitro*.

In the beta diversity cluster analysis, the abundance of the dominant genus, *Prevotella*, was similar in the low-dose 5-HTP groups (2 mg/kg and 4 mg/kg) at 12 h (**Figure 2A**). Additionally, its abundance was similar in the high-dose 5-HTP groups (8 mg/kg and 10 mg/kg) at 12 h. However, the abundance of *Succinivibrio* in the high-dose 5-HTP groups (8 mg/kg and 10 mg/kg) was significantly higher at 12 h. Furthermore, at 12 h, the amount of *Prevotella* in the high-dose 5-HTP groups (8 mg/kg and 10 mg/kg) was lower than that in the low-dose 5-HTP groups (2 mg/kg and 4 mg/kg), indicating that the high-dose 5-HTP groups (8 mg/kg and 10 mg/kg) increased the amount of *Succinivibrio* and reduced the amount of *Prevotella*. At 48 h, the high-dose 5-HTP groups (8 mg/kg and 10 mg/kg) showed significantly increased *Succinivibrio* abundance, suggesting that the addition of 5-HTP successfully increased *Succinivibrio* abundance (**Figure 2B**).

### 3.5 Rumen microbial metabolic pathway analysis *in vitro*

The KEGG enrichment pathways of 5-HTP supplementation, which mimicked rumen fermentation microorganisms *in vitro* are shown in **Figure 2C**. These pathways fall into seven level-1 categories: biosynthesis, degradation/utilization/absorption, disinfection, precursor metabolites and energy production, glycan pathways, polymer modifications, and metabolic clusters. Among these, the biosynthetic pathway was the dominant metabolic pathway.

### 3.6 Rumen metabolite *in vitro*

**Figure 3:**
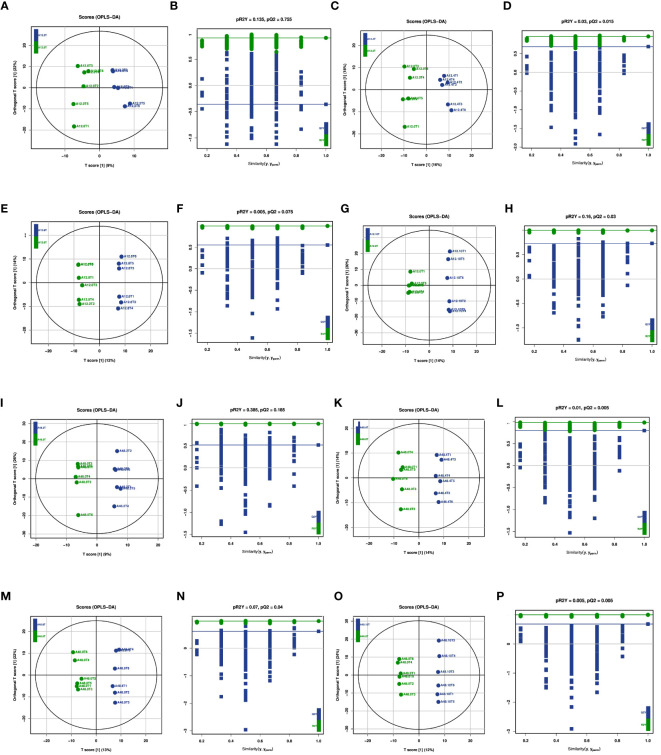
OPLS-DA score and permutations of metabolites under 5-HTP supplementation at different fermentation times *in vitro* **(A)** OPLS-DA score between A12.0T and A12.2T; **(B)** Permutations between A12.0T and A12.2T; **(C)** OPLS-DA score between A12.0T and A12.4T; **(D)** Permutations between A12.0T and A12.4T; **(E)** OPLS-DA score between A12.0T and A12.8T; **(F)** Permutations between A12.0T and A12.8T; **(G)** OPLS-DA score between A12.0T and A12.10T; **(H)** Permutations between A12.0T and A12.10T; **(I)** OPLS-DA score between A48.0T and A48.2T; **(J)** Permutations between A48.0T and A48.2T; **(K)** OPLS-DA score between A48.0T and A48.4T; **(L)** Permutations between A48.0T and A48.4T; **(M)** OPLS-DA score between A48.0T and A48.8T; **(N)** Permutations between A48.0T and A48.8T; **(O)** OPLS-DA score between A48.0T and A48.10T; **(P)** Permutations between A48.0T and A48.10T) A total of 10 sets of experiments, each group of 6 parallel samples, the total number of samples n=60.) Among them, A12.0T, A12.2T, A12.4T, A12.8T, and A12.10T indicated fermentation for 12 h, and 0, 2, 4, 8, and 10 mg/kg 5-HTP additives were added, respectively. A48.0T, A48.2T, A48.4T, A48.8T, and A48.10T indicated fermentation for 48 h, and 0, 2, 4, 8, and 10 mg/kg 5-HTP additives were added, respectively.

The OPLS-DA model was used to compare and analyze rumen metabolites, and its suitability was evaluated through a permutation test. As indicated in **Figures 3A, C, E, G**, 5-HTP had distinct effects on rumen metabolites compared with the 0 mg/kg 5-HTP group at 12 h. Similarly, at 48 h, there was a significant disparity between the groups (**Figures 3I, K, M, O**). These results collectively suggest that 5-HTP has significant effects on rumen metabolites. Furthermore, the 200 permutation test models in each group did not overfit, confirming the reliability of the models and experimental results (**Figures 3B, D, F, H, J, L, N, P**).

### 3.7 Differential metabolites screening and analysis *in vitro*

**Figure 4:**
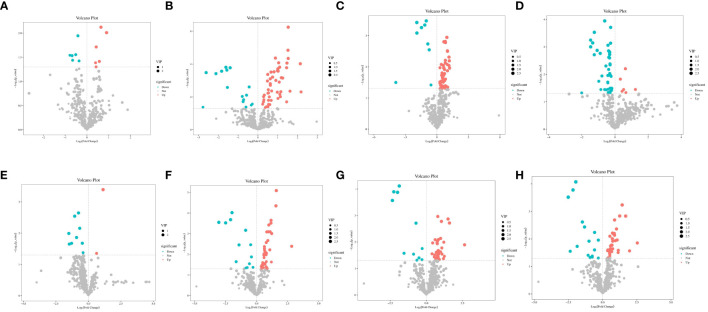
Volcano plot of differential metabolites under 5-HTP supplementation at different fermentation times *in vitro* **(A)** A12.0T and A12.2T intergroup volcanoes; B: A12.0T and A12.4T intergroup volcanoes; **(C)** A12.0T and A12.8T intergroup volcanoes; **(D)** A12.0T and A12.10T intergroup volcanoes; **(E)** A48.0T and A48.2T intergroup volcanoes; **(F)** A48.0T and A48.4T intergroup volcanoes; **(G)** A48.0T and A48.8T intergroup volcano plot; **(H)** A48.0T and A48.10T intergroup volcano plot).

**Figure 5:**
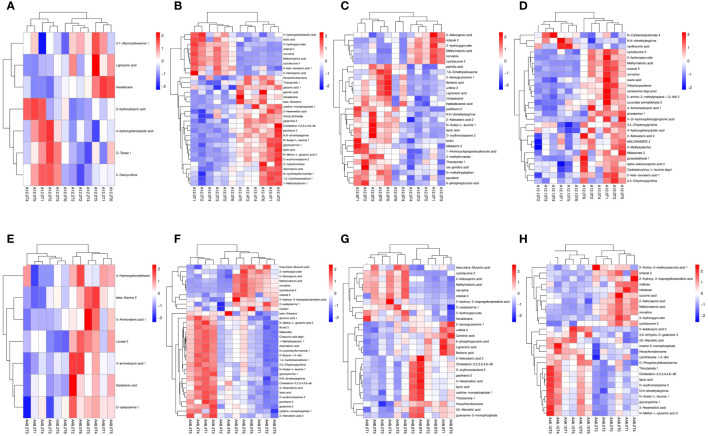
Heatmap analysis of differential metabolites under 5-HTP supplementation at different fermentation times *in vitro* **(A)** heat map of differential metabolites between A12.0T and A12.2T; B: A12.0T and A12.4T; **(C)** A12.0T and A12.8T; **(D)** A12.0T and A12.10T; **(E)** A48.0T and A48.2T; **(F)** A48.0T and A48.4T; **(G)** A48.0T and A48.8T; **(H)** A48.0T and A48.10T). A total of 10 sets of experiments, each group of 6 parallel samples, the total number of samples n = 60. Among them, A12.0T, A12.2T, A12.4T, A12.8T, and A12.10T indicated fermentation for 12 h, and 0, 2, 4, 8, and 10 mg/kg 5-HTP supplemented, respectively. A48.0T, A48.2T, A48.4T, A48.8T, and A48.10T indicated fermentation for 48 h, and 0, 2, 4, 8 and 10 mg/kg 5-HTP supplemented, respectively.

Differential metabolites were identified using the OPLS-DA model and screened based on variable importance projection (VIP) values > 1 and *P* < 0.05. **Supplementary Table S3** presents details on the differential metabolites identified in each group. We identified significant differences in metabolites using volcano plots (**Figure 4**). Red represents significantly upregulated metabolites, blue represents significantly downregulated metabolites, and gray represents no significant differences. The colors distinguish the level of metabolite richness within a row, with red denoting high metabolite richness and blue denoting low metabolite richness. A total of seven differential metabolites were identified between A12.0T and A12.2T. Among these, 4’, 7-dihydroxy flavanone 1, tetracosanoic acid and hexadecane contents were higher in rumen fluid metabolites supplemented with 2 mg/kg 5-HTP group (**Figure 5A**). The A12.0T and A12.4T groups had 32 distinct metabolites (*P* < 0.05) (**Figure 5B**). Of these, octanal 2 and 3-hydroxy pyruvate were lower in rumen fluid metabolites with 4 mg/kg 5-HTP, whereas other metabolite amounts were higher. Between the A12.0T and A12.8T groups, 29 different metabolites (*P* < 0.05) were selected (**Figure 5C**). The 0 mg/kg 5-HTP group contained high amounts of octanal 2, 3-hydroxy pyruvate, and methylmalonic acid. A total of 27 unique metabolites (*P* < 0.05) were selected between the A12.0 T and A12.10 T groups, most of them abundantly present in rumen fluid supplemented with 10 mg/kg 5-HTP (**Figure 5D**). Seven differential metabolites were identified in the A48.0T and A48.2T groups (**Figure 5E**). These seven differential metabolites were present in lower amounts in the 2 mg/kg 5-HTP supplemented group after 48 h (*P* < 0.05). In the A48.0T and A12.4T groups, 33 differential metabolites (*P* < 0.05) were identified (**Figure 5F**). Specifically, 1-methyl hydantoin 1; 1,3-cyclohexanedione 1; and lipoic acid were more abundant in the 4 mg/kg 5-HTP group. Twenty-seven differential metabolites were identified in the A48.0T and A48.8T groups (**Figure 5G**). Among these, 2-oxovaleric acid, panthenol 2, lipoic acid, and 3-hexanedioic acid were highly abundant in the 8 mg/kg 5-HTP group. In total, 27 differential metabolites (*P* < 0.05) were identified between A48.0T and A48.10T groups (**Figure 5H**). Among these, metabolites such as 2-hexanoic acid, N-acetyl-L-leucine 1, and N, N-dimethylarginine were found to be low in the 0 mg/kg 5-HTP group, whereas the other metabolites were low in the 10 mg/kg 5-HTP group. These results indicated that different doses of 5-HTP led to significant changes in rumen metabolite content. Further analysis, based on a fold difference of more than 2 or less than 0.5 and a P-value of less than 0.01, revealed 15 different metabolites, including three alcohols, nine organic acids, one carbohydrate, one nucleoside, and one aldehyde. In the high-dose 5-HTP groups (8 mg/kg and 10 mg/kg), three differential metabolites: uridine 2, lignoceric acid, and N-methyltryptophan were identified, while the low-dose 5-HTP groups (2 mg/kg and 4 mg/kg) showed 6 separate metabolites, including panthenol 2, lipoic acid, β-sitosterol, gluconic acid, N,N-dimethylarginine, and cholesterol-2,2,3,4,4,6-d6. Norvaline, cycloleucine 2, octanal 2, and methylmalonic acid were the four common differential metabolites of 5-HTP in both the low- and high-dose groups.

### 3.8 Differential metabolite pathway enrichment analysis *in vitro*

**Figure 6:**
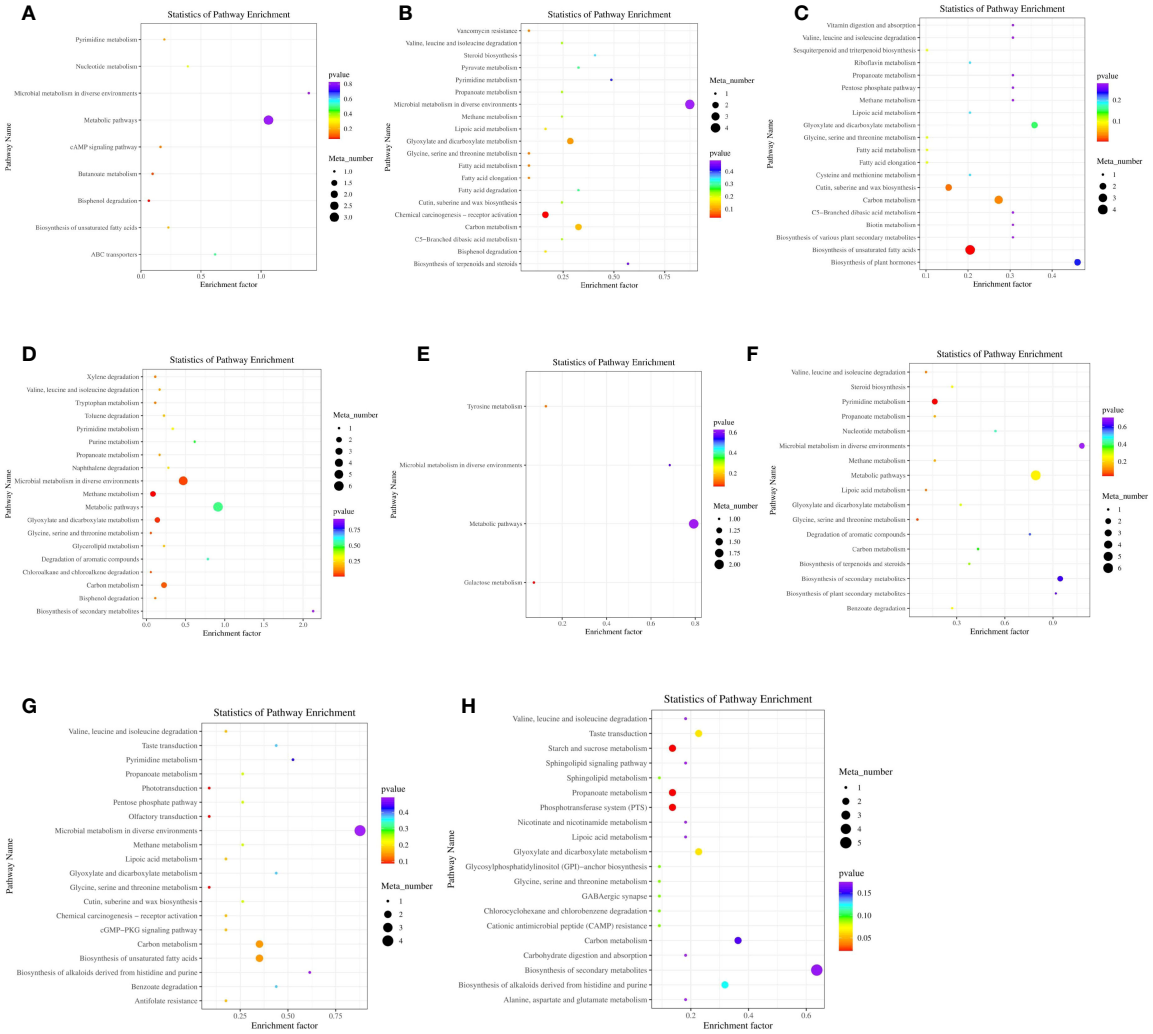
Differential metabolite KEGG enrichment analysis under 5-HTP supplementation at different fermentation times *in vitro* **(A)** A12.0T and A12.2T differential metabolite pathway enrichment plot; **(B)** A12.0T and A12.4T; **(C)** A12.0T and A12.8T; **(D)** A12.0T and A12.10T group; **(E)** A48.0T and A48.2T group; **(F)** A48.0T and A48.4T groups; **(G)** A48.0T and A48.8T groups; **(H)** A48.0T and A48.10T groups). A total of 10 sets of experiments, each group of 6 parallel samples, the total number of samples n = 60.) Among them, A12.0T, A12.2T, A12.4T, A12.8T and A12.10T indicated fermentation for 12 h, and 0, 2, 4, 8 and 10 mg/kg 5-HTP supplemented, respectively. A48.0T, A48.2T, A48.4T, A48.8T, and A48.10T indicated fermentation for 48 h, and 0, 2, 4, 8 and 10 mg/kg 5-HTP supplemented, respectively.

Differential metabolites were analyzed using KEGG metabolic pathway analysis (**Figure 6**; **Supplementary Table S4**). A total of 87 metabolic pathways were enriched, and 36 metabolites were annotated. **Figure 6A** indicated 9 metabolic pathways that were rich in different metabolites between the A12.0T and A12.2T groups. **Figure 6B** shows that 20 metabolic pathways were mainly enriched for differential metabolites between the A12.0T and A12.4T groups, with 11 annotated metabolites involving multiple pathways. Twenty metabolic pathways, mainly enriched in differential metabolites between the A12.0T and A12.8T groups are shown in **Figure 6C**, involving 12 annotated metabolites of multiple pathways. **Figure 6D** shows that 19 metabolic pathways were enriched in the differential metabolites between the A12.0T and A12.10T groups. **Figure 6E** shows 4 metabolic pathways that were primarily enriched in the differential metabolites between the A48.0T and A48.2T groups, with two annotated metabolites involved in multiple pathways. As shown in **Figure 6F**, 17 metabolic pathways were enriched in the differential metabolites between the A48.0T and A48.4T groups, with six annotated metabolites involving multiple pathways. **Figure 6G** shows 20 metabolic pathways that were mainly enriched in differential metabolites between the A48.0T and A48.8T groups, with nine annotated metabolites involving multiple pathways. Finally, **Figure 6H** shows 20 metabolic pathways, mostly composed of different metabolites between the A48.0T and A48.10T groups, and nine annotated metabolites involved in multiple pathways.

The low-dose 5-HTP groups (2 mg/kg and 4 mg/kg) were screened for two metabolic pathways: chemical carcinogenesis receptor activation and pyrimidine metabolism. In the high-dose 5-HTP groups (8 mg/kg and 10 mg/kg), 9 metabolic pathways were screened, including biosynthesis of unsaturated fatty acids, methane metabolism, cutin, suberin, wax biosynthesis, carbon metabolism, glyoxylate and dicarboxylate metabolism, microbial metabolism in different environments, propionate metabolism, starch and sucrose metabolism, and the phosphotransferase system (starch and sucrose metabolism).

### 3.9 Rumen metabolite panel and microbial correlation analysis *in vitro*

**Figure 7:**
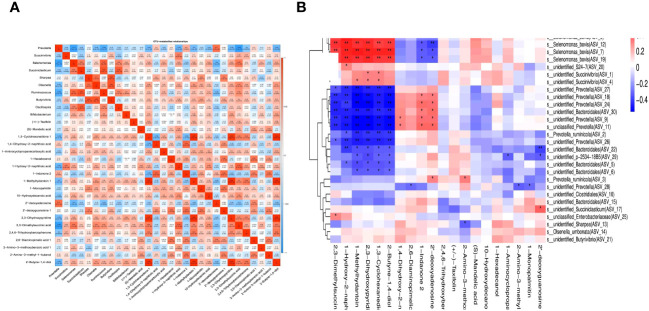
Rumen differential metabolite correlated heat map with bacteria at genus-level **(A)** and Spearman-related heat map of rumen differential metabolites and ASV **(B)** under 5-HTP supplementation at different fermentation times *in vitro*. The abscissa represents the differential metabolite, the ordinate represents the relative abundance of ASV of top 30 (the left side represents the species with the highest classification of this ASV), the “*” and “**” signs in the square represent the *P* ≤ 0.05, and *P* ≤ 0.01, respectively.

Pearson’s correlation coefficients and correlation heat maps were used to examine the relationships between various metabolites and bacterial genera (**Figure 7A**). The results showed positive correlations between different metabolites and bacterial genera, including 2’-deoxyadenosine with *Prevotella*, 1-hydroxy-2-naphthoic acid, with *Succinivibrio*, *Selenomonas* with *Ruminococcus*, *Succiniclasticum* with *Oscillospira*, 1-monopalmitin with *Butyrivibrio*, and *Sharpea*, 1-hydroxylamine, 2-methylnaphthyl acid, and 1-monopalmitin. Additionally, there was a positive correlation between 3-cyclohexanedione 1, 2,3-Dihydroxypyridine, and 1-methylhydantoin1, while 2-butyne-1,4-diol positively correlated with 1,3-cyclohexanedione 1, 1-methylhydantoin1, and 2,3-dihydroxypyridine. Despite these correlations, most differentially expressed metabolites were negatively correlated with the *Prevotella* genus.

Spearman’s correlation coefficient was used to investigate the relationship between differential rumen metabolites and their correlation with ASV (**Figure 7B**). This analysis sought to comprehend the interaction between metabolites and microorganisms, specifically focusing on the impact of metabolites on bacterial composition and the mechanism of changes in flora in complex diseases. Correlation coefficients were computed based on the top 30 bacteria and differential metabolites in relation to the relative abundance of ASV using the psych package in R. The findings revealed a positive correlation between rumen differential metabolites and ASV and vice versa.

### 3.10 Growth performance of sheep

**Table 3** shows the effects of 5-HTP on body weight, average daily gain (ADG), and DMI. The 5-HTP group consumed 0.01 kg more dry matter than the control group during days 0-60 (*P* > 0.05). On day 30, the sheep in the 5-HTP group weighed 2.4 kg more on average compared to the control group (*P* < 0.05). In the 60-day experiment, the 5-HTP group gained an average of 17.7 kg, slightly more than the control group’s 16.5 kg. The 5-HTP group consistently had higher daily weight gain values at each stage, but the differences were not statistically significant (*P* > 0.05).

**Table 3:** Effects of 5-HTP on dry matter intake, body weight gain, and average daily gain in sheep.

Item	Days	Control	5-HTP group	t	*P*-value
Dry matter intake (kg/d)	0	1.09 ± 0.01	1.10 ± 0.00	1.00	0.37
	15	1.37 ± 0.01	1.38 ± 0.00	1.00	0.37
	30	1.61 ± 0.01	1.63 ± 0.00	1.16	0.30
	45	1.9 ± 0.00	1.9 ± 0.00	0.00	1.00
	60	1.45 ± 0.01	1.46 ± 0.00	1.00	0.19
Body weight (kg)	0	19.50 ± 0.63	20.70 ± 0.46	-1.53	0.17
	15	23.6 ± 0.64	25.00 ± 0.35	1.91	0.09
	30	27.7 ± 0.64^b^	30.10 ± 0.51^a^	2.92	<0.05
	45	32.1 ± 1.01	33.70 ± 0.56	1.34	0.22
	60	36 ± 1.00	38.40 ± 1.16	1.57	0.16
Average daily gain (kg/d)	0-15	0.27 ± 0.01	0.29 ± 0.01	-0.89	0.40
	15-30	0.27 ± 0.03	0.34 ± 0.02	-2.02	0.08
	30-45	0.29 ± 0.04	0.24 ± 0.01	1.17	0.28
	45-60	0.26 ± 0.02	0.31 ± 0.04	-1.10	0.30
	0-60	0.28 ± 0.01	0.30 ± 0.01	1.60	0.15
Total body weight gain (kg)	60	16.5 ± 0.45	17.70 ± 0.8	-1.31	0.23

^a,b^Different superscripts in the same raw implies their mean values are significantly different (P ≤ 0.05).

### 3.11 Sheep blood indices

**Table 4** shows that the plasma INS level in the 5-HTP group was higher than that in the control at 60 days (*P* < 0.05). There were no significant differences in the T-AOC, MDA, SOD, GSH-PX, or CAT between the two groups (*P* > 0.05). The control group showed significantly higher total protein levels on days 15, 30, 45 (*P* < 0.05), and 60 (*P* < 0.01). On day 15, the 5-HTP group showed significantly higher albumin levels (*P* < 0.05). Globulin levels were higher in the control group on days 15, 30 (*P* < 0.05), and 60 (*P* < 0.01). Low-density lipoprotein levels were higher in the control group on day 30 (*P* < 0.05). On day 15, the urea concentration in the 5-HTP group was higher than that in the control group (*P* < 0.05, **Table 5**).

**Table 4:** Effects of 5-HTP on blood antioxidant levels in sheep.

Item	Day	Control group	5-HTP group	t	*P*-value
Antioxidant indices					
T-AOC (U/mL)	0	7.86 ± 0.31	7.68 ± 0.21	0.47	0.65
	15	8.53 ± 0.31	8.31 ± 0.12	0.68	0.52
	30	9.13 ± 0.25	9.66 ± 0.21	-1.64	0.14
	45	10.59 ± 0.49	10.89 ± 0.49	-0.44	0.67
	60	10.50 ± 0.48	10.06 ± 0.85	0.45	0.66
MDA (nmol/mL)	0	6.50 ± 0.45	6.42 ± 0.18	0.17	0.87
	15	5.21 ± 0.14	5.16 ± 0.30	0.162	0.88
	30	3.83 ± 0.29	4.68 ± 0.37	-1.78	0.11
	45	3.12 ± 0.37	2.90 ± 0.27	0.49	0.63
	60	3.27 ± 0.11	3.76 ± 0.31	-1.52	0.19
SOD (U/mL)	0	44.63 ± 4.50	46.34 ± 2.58	-0.33	0.75
	15	53.94 ± 3.40	51.41 ± 1.88	0.65	0.53
	30	61.75 ± 1.74	61.17 ± 0.45	0.32	0.76
	45	76.27 ± 2.69	68.53 ± 7.01	1.03	0.33
	60	68.26 ± 2.06	64.17 ± 3.08	1.10	0.30
GSH-PX (U/mL)	0	281.83 ± 15.62	322.98 ± 21.39	-1.55	0.16
	15	330.79 ± 22.15	329.52 ± 22.86	0.04	0.97
	30	382.69 ± 30.58	422.76 ± 23.16	-1.05	0.33
	45	500.60 ± 15.85	525.67 ± 20.16	-0.98	0.36
	60	426.99 ± 7.08	468.10 ± 17.88	-2.14	0.07
CAT (U/mL)	0	29.71 ± 1.23	31.63 ± 1.27	-1.09	0.31
	15	31.82 ± 1.88	34.32 ± 1.79	-0.97	0.36
	30	36.69 ± 1.09	36.16 ± 0.35	0.47	0.66
	45	46.89 ± 4.61	52.95 ± 7.40	-0.70	0.51
	60	39.42 ± 1.81	42.00 ± 2.37	-0.87	0.41
Blood hormone					
INS (μIU/mL)	0	13.86 ± 0.20	14.67 ± 0.50	-1.39	0.20
	15	13.66 ± 0.56	13.14 ± 0.31	0.80	0.45
	30	12.08 ± 0.71	13.88 ± 1.15	-1.32	0.22
	45	16.16 ± 0.85	15.51 ± 0.61	0.62	0.56
	60	12.35 ± 0.35^b^	15.59 ± 1.06^a^	-2.90	<0.05
GH (ng/mL)	0	4.40 ± 0.19	4.41 ± 0.26	0.02	0.98
	15	4.59 ± 0.16	5.55 ± 0.59	1.56	0.16
	30	5.61 ± 0.32	5.78 ± 0.29	0.41	0.69
	45	6.81 ± 0.56	7.54 ± 0.34	1.13	0.29
	60	6.22 ± 0.24	6.71 ± 0.28	1.33	0.22

^a,b^Different superscripts in the same raw implies their mean values are significantly different (P ≤ 0.05). T-AOC, total antioxidant capacity; MDA, malondialdehyde; SOD, superoxide dismutase; GSH-PX, glutathione peroxidase; CAT, catalase; INS, insulin; GH, growth hormone.

**Table 5:** Effects of 5-HTP on blood biochemical indices in sheep.

Item	Day	Control group	5-HTP group	t	*P*-value
TP (g/L)	0	64.60 ± 1.57	71.36 ± 2.69	-2.17	0.06
	15	76.00 ± 2.06^a^	67.87 ± 1.86^b^	2.93	<0.05
	30	80.13 ± 3.16^a^	64.38 ± 4.45^b^	2.89	<0.05
	45	90.36 ± 4.47^a^	76.87 ± 1.85^b^	2.79	<0.05
	60	76.80 ± 1.34^A^	65.05 ± 2.04^B^	4.81	<0.01
ALB (g/L)	0	24.01 ± 1.91	25.02 ± 1.01	-0.47	0.65
	15	24.91 ± 1.37^b^	27.19 ± 0.82^a^	2.93	<0.05
	30	26.66 ± 1.22	26.15 ± 1.11	0.31	0.76
	45	26.80 ± 1.50	24.57 ± 1.91	0.92	0.39
	60	26.90 ± 2.09	30.15 ± 0.66	-1.49	0.18
GLB (g/L)	0	40.60 ± 1.98	46.34 ± 3.17	-1.54	0.16
	15	51.09 ± 2.49^a^	40.67 ± 1.32^b^	3.69	<0.05
	30	53.47 ± 2.30^a^	38.23 ± 4.59^b^	2.97	<0.05
	45	63.57 ± 4.12	52.30 ± 3.44	2.10	0.07
	60	49.90 ± 1.80^A^	34.90 ± 2.44^B^	4.95	<0.01
TC (mmol/L)	0	1.26 ± 0.21	0.96 ± 0.92	1.29	0.23
	15	1.05 ± 0.15	1.14 ± 0.13	-0.45	0.66
	30	1.30 ± 0.09	1.20 ± 0.07	0.82	0.44
	45	1.80 ± 0.31	1.12 ± 0.41	1.31	0.23
	60	1.49 ± 0.08	1.42 ± 0.14	0.39	0.71
TG (mmol/L)	0	0.35 ± 0.01	0.33 ± 0.04	0.28	0.79
	15	0.38 ± 0.07	0.32 ± 0.05	0.77	0.47
	30	0.37 ± 0.05	0.22 ± 0.04	2.36	0.05
	45	0.82 ± 0.09	0.50 ± 0.15	1.80	0.11
	60	0.37 ± 0.03	0.39 ± 0.06	-0.27	0.79
HDL (mmol/L)	0	0.71 ± 0.13	0.60 ± 0.09	0.71	0.50
	15	0.62 ± 0.07	0.67 ± 0.05	-0.53	0.61
	30	0.71 ± 0.04	0.61 ± 0.06	1.31	0.23
	45	0.96 ± 0.05	0.85 ± 0.09	1.08	0.31
	60	0.84 ± 0.06	0.68 ± 0.06	1.81	0.12
LDL (mmol/L)	0	0.30 ± 0.07	0.33 ± 0.03	-0.36	0.73
	15	0.77 ± 0.19	0.62 ± 0.11	0.68	0.52
	30	0.82 ± 0.06^a^	0.61 ± 0.03^b^	3.50	<0.05
	45	1.00 ± 0.10	0.87 ± 0.04	1.14	0.30
	60	0.56 ± 0.04	0.64 ± 0.12	-0.62	0.56
GLU (mmol/L)	0	3.85 ± 0.37	3.28 ± 0.18	1.39	0.22
	15	3.02 ± 0.35	3.49 ± 0.22	-1.13	0.29
	30	3.25 ± 0.24	3.22 ± 0.14	0.11	0.92
	45	4.18 ± 0.35	4.00 ± 0.15	0.45	0.67
	60	5.19 ± 0.10	4.18 ± 0.41	2.39	0.07
UREA (mmol/L)	0	6.39 ± 0.52	4.92 ± 0.55	1.93	0.09
	15	3.05 ± 0.31^b^	4.85 ± 0.43^a^	-3.39	<0.05
	30	4.05 ± 0.76	4.01 ± 0.53	0.05	0.97
	45	4.33 ± 0.69	5.55 ± 0.79	-1.16	0.28
	60	6.54 ± 1.04	4.05 ± 0.32	2.28	0.05
NEFA (mmol/L)	0	0.09 ± 0.01	0.15 ± 0.03	-1.98	0.08
	15	0.11 ± 0.04	0.11 ± 0.01	0.09	0.93
	30	0.19 ± 0.03	0.14 ± 0.04	0.88	0.40
	45	0.59 ± 0.09	0.46 ± 0.12	0.90	0.40
	60	0.16 ± 0.07	0.27 ± 0.06	-1.16	0.30

^a,b^Different superscripts in the same raw implies their mean values are significantly different (P ≤ 0.05). ^A,B^Different superscripts in the same raw implies their mean values are significantly different (P ≤ 0.01). TP, total protein; ALB, albumin; GLB, globulin; TC, total cholesterol; TG, triglyceride; HDL, high-density lipoprotein; LDL, low-density lipoprotein; GLU, glucose; NEFA, non-esterified fatty acids.

### 3.12 Blood 5-HTP, 5-HT, MT content in sheep

**Table 6** shows the initial increase and subsequent decline in the blood levels of 5-HTP, 5-HT, and MT. The concentrations increased slowly during the first 15 days, peaked in both groups on the 45^th^ day, and then decreased on the 60^th^ day. There were no significant differences in the concentration of 5-HTP, 5-HT, or MT between the two groups (*P* > 0.05). However, the levels of 5-HTP and 5-HT were higher in the 5-HTP group compared to the control group on days 15, 30, and 60. Additionally, sheep supplemented with 8 mg/kg 5-HTP showed higher MT levels on days 15-60 compared to the control group.

**Table 6:** Effects of 5-HTP on blood 5-HTP, 5-HT, MT content in sheep.

Item	Day	Control group	5-HTP group	t	*P*-value
5-HTP (ng/mL)	0d	166.99 ± 10.81	156.64 ± 6.73	0.81	0.44
	15d	165.05 ± 4.17	172.09 ± 4.95	-1.09	0.31
	30d	208.82 ± 5.02	211.74 ± 4.74	-0.42	0.68
	45d	254.25 ± 13.62	252.55 ± 11.31	0.10	0.93
	60d	243.41 ± 11.79	249.04 ± 8.07	-0.39	0.70
5-HT (ng/mL)	0d	115.98 ± 6.43	113.31 ± 2.30	0.39	0.71
	15d	114.23 ± 4.10	114.90 ± 3.51	-0.12	0.90
	30d	128.14 ± 3.62	132.84 ± 14.94	-0.31	0.77
	45d	197.28 ± 7.68	196.58 ± 5.80	0.07	0.94
	60d	172.88 ± 10.41	181.87 ± 4.21	-0.80	0.45
MT (pg/mL)	0d	33.92 ± 1.36	32.78 ± 1.88	0.49	0.64
	15d	39.43 ± 2.39	40.63 ± 1.55	-0.42	0.69
	30d	41.71 ± 1.37	42.70 ± 0.63	-0.66	0.54
	45d	48.92 ± 1.80	53.05 ± 3.15	-1.14	0.29
	60d	43.39 ± 2.93	47.67 ± 1.87	-1.23	0.25

5-HTTP, hydroxytryptophan; 5-HT, hydroxytryptamine; MT, melatonin.

### 3.13 Blood metabolites in sheep

**Figure 8:**
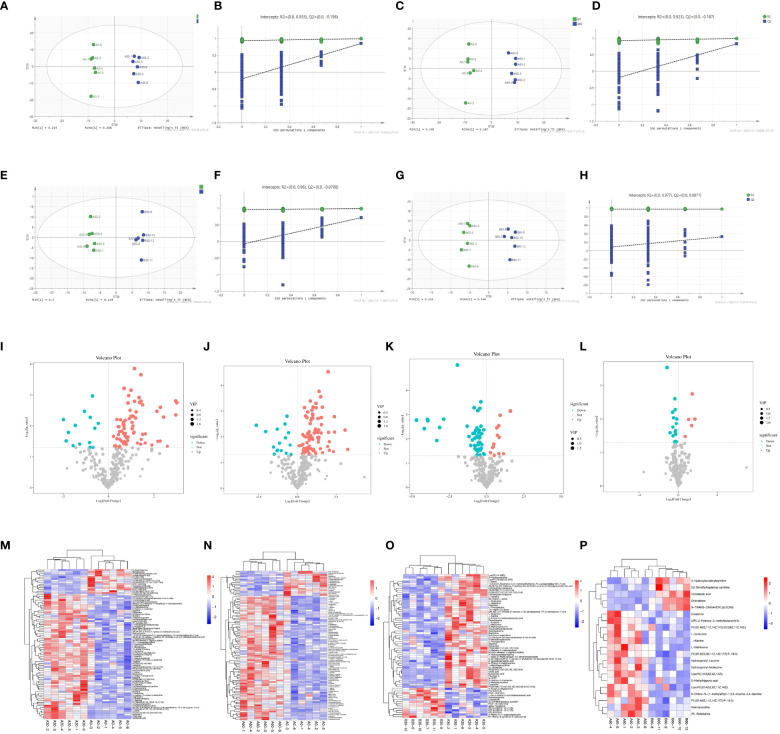
OPLS-DA score and replacement test plot of blood metabolite in sheep **(A)** OPLS-DA score plot between A0 and A30 groups; **(B)** Test chart of intergroup displacement between A0 and A30; **(C)** OPLS-DA score plot between A0 and A60 groups; **(D)** A0 and A60 intergroup displacement test chart; **(E)** OPLS-DA score plot between A30 and B30 groups; **(F)** A30 and B30 intergroup displacement test chart; **(G)** OPLS-DA score plot between A60 and B60 groups; **(H)** A60 and B60 intergroup displacement test chart); volcanic plot of differential metabolite screening of sheep blood **(I)** A0 and A30 intergroup volcanic plot; **(J)** A0 and A60 intergroup volcano map; **(K)** A30 and B30 inter-group volcano map; **(L)** A60 and B60 inter-group volcano map) and Horizontal clustering plot of differential metabolites in sheep **(M)** A0 and A30 hierarchical clustering plot; **(N)** A0 and A60 groups; **(O)** A30 and B30; **(P)** A60 and B60).

The samples were classified and identified using an orthogonal partial least-squares discriminant analysis (OPLS-DA) model. The scatter plot of the OPLS-DA score for each sample indicates that the comparison between the two groups was highly significant. All samples fell within the 95% confidence interval and there were noticeable differences among the four groups (A30 vs. A0, A60 vs. A0, A30 vs. B30, and A60 vs. B60) based on the scores of the main components shown in **Figures 8A, C, E, G**. Differences among the groups were also observed based on the score of the orthogonal component on the ordinate (t [1]) o) (**Figures 8B, D, F, H**). To assess overfitting, a permutation test was conducted on the sequencing data of the OPLS-DA model. The results of the 200 substitution test models in each group indicated no overfitting, suggesting that the above models and experimental results were accurate.

### 3.14 Screening and analysis of significant differential metabolites in sheep

The OPLS-DA model used multivariate analysis to combine variable importance projection (VIP) values > 1 with Student’s t-test (P < 0.05) for each metabolite between two groups to initially screen for differential metabolites (27). Therefore, 84, 94, 66, and 20 differential metabolites were screened for the A0 and A30, A0 and A60, A30 and B30, and A60 and B60 treatments, respectively. Volcano plots displayed various metabolites, with red indicating significant upregulation, blue indicating significant downregulation, and gray indicating no significant differences (**Figures 8I, J, K, L**). Hierarchical clustering analysis presented in a clustered heatmap revealed significant differential metabolites in the blood, with red representing high metabolite richness and blue representing low metabolite richness. The 5-HTP group exhibited higher levels of nutritional cholic acid, flavonol A, and 5-HEPE than the control group (**Figure 8M**). Additionally, the abundance of trimethylamine N-oxide, 3-hydroxyisovalylcarnitine, and fragransol B in the 5-HTP group was higher than that in the control group (**Figure 8N**). Furthermore, the abundance of 3-indadiene, acetonitrile, L-valine, and L-norleucine in the 5-HTP group was higher (**Figure 8O**). Finally, the levels of the differential metabolites (R-Pelletierine, homoarecoline, L-Methionine, creatinine, and hydroxyprolyl-leucine) between A60 and B60 were higher in the 5-HTP group than in the control group (**Figure 8P**).

### 3.15 Significant differential metabolite pathway enrichment analysis in sheep

**Figure 9:**
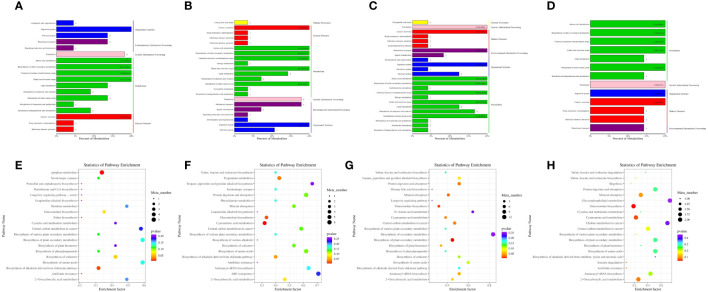
KEGG enrichment analysis of blood metabolite in sheep **(A)** A0 and A30 intergroup pathway type analysis diagram; **(B)** Analysis of pathway types between A0 and A60 groups; **(C)** A30 and B30; **(D)** between A60 and B60) and KEGG enrichment analysis plot of different compounds between different groups € KEGG enrichment analysis plot between A0 and A30 groups; **(F)** A0 and A60 groups; **(G)** A30 and B30 groups; **(H)** A60 and B60 groups).

We used the KEGG pathway database (28, 29) (Figure www.kegg.jp/kegg/pathway.html) to annotate the differential metabolites for corresponding metabolic pathways. Differential metabolite pathway analysis maps of the different groups have been presented in **Figures 9A–D**. There were significant differences in the metabolites between the A0 and A30 groups, resulting in 18 KEGG functional clusters at the secondary functional classification level. The dominant clusters were the digestive system and amino acid metabolites. Twenty-two KEGG functional clusters were obtained between the A0 and A60 groups, with a cancer overview and dominant amino acid metabolism. A total of 24 KEGG functional clusters were obtained between the A30 and B30 groups, with dominant amino acid metabolism. Thirteen KEGG functional clusters with dominant amino acid metabolism and an overview of cancer were obtained between the A60 and B60 groups. Global and overview maps of the KEGG secondary pathways were dominant among all four pairs of groups. Further metabolic pathway analysis is needed to identify critical pathways with strong connections to metabolite differences, as KEGG annotation analysis only identifies pathways involving all differential metabolites (30). As shown in **Figure 9**, there were significantly different metabolites between groups A0 and A30 (**Figure 9E**), which were mainly enriched in the cofactor biosynthesis, tryptophan amino acid metabolism, amino acid synthesis, and plant secondary metabolite synthesis pathways. Significantly differential metabolites between the A0 and A60 groups (**Figure 9F**) were mainly enriched in the biosynthesis of amino acids, protein digestion and absorption, mineral absorption, and cofactor biosynthesis in cancer. Protein digestion and absorption, amino acid biosynthesis, plant secondary metabolite production, and mineral absorption were the key areas with significantly different metabolites between the A30 and B30 groups (**Figure 9G**). Significantly different metabolites in the A60 and B60 groups (**Figure 9H**) were mainly enriched for aminoacyl-tRNA biosynthesis, mineral absorption, 2-oxocarboxylic acid metabolism, and protein digestion.

## 4 Discussion

### 4.1 Effects of 5-HTP supplementation on rumen fermentation parameters *in vitro*

The rumen pH remained within the normal range of 6.0 to 6.8. The addition of 5-HTP did not cause a significant change in the pH level (*P* > 0.05). However, the pH levels decreased in both groups as fermentation progressed, likely due to carbohydrate fermentation by microorganisms. This is consistent with the finding that VFA accumulation in fermentation flasks leads to a decrease in pH (31). The optimal level of NH_3_-N for microbial growth is between 0.35-29 mg/dL (32), with higher concentrations beneficial for cellulolytic bacteria growth and DM degradation. In our study, the NH_3_-N concentrations ranged from 14.11 to 71.67 mg/dL, with a significant increase (*P* < 0.05) after 12 h. As fermentation time increased, the pH of the rumen fluid decreased, hindering the growth of structural carbohydrate-decomposing bacteria and increasing the production of NH_3_-N. The pH decrease was significantly influenced by the 5-HTP dose (*P* < 0.05) and the interaction (*P* < 0.01). The low-dose 5-HTP groups (2 mg/kg and 4 mg/kg) had higher NH_3_-N concentrations before 24 h, whereas the high-dose groups (8 mg/kg and 10 mg/kg) had higher concentrations after 24 h. Rumen microorganisms ferment carbohydrates to produce acetic, propionic, and butyric acids, providing 70-80% of the energy needs and 95% of the total fatty acid content of ruminants (33). In our study, acetic acid was the primary component that dominated the TVFA content. Both acetic and propionic acids exhibited similar trends, with higher acid production in the high-dose groups (8 mg/kg and 10 mg/kg). This is consistent with the observed decrease in pH and increase in NH_3_-N concentration. High doses of 5-HTP (8 mg/kg and 10 mg/kg) can enhance *in vitro* VFA fermentation and improve rumen fermentation efficiency. Specifically, the 8 mg/kg group had higher levels of TVFA, propionic acid, and acetic acid compared to the 10 mg/kg group. The DMD rate is also an important metric for evaluating feed value, reflecting the digestion ability of ruminants (34). The group that received 8 mg/kg of 5-HTP showed a higher DMD rate at 24 h, indicating increased microbial activity and improved fermentation. Ruminants produce a significant amount of gas during feed fermentation by rumen microorganisms, which reflects the fermentation status of the feed (35). Our study found that 5-HTP had a significant effect on gas production (*P* < 0.05). The group that received 8 mg/kg of 5-HTP showed higher gas production at 12, 24, and 48 h and a higher DMD rate at 24 h. These suggest that 5-HTP can enhance carbohydrate digestion, increase DMD rate, and organic acid production.

### 4.2 Effects of 5-HTP on rumen microbial community composition *in vitro*

The low-dose 5-HTP groups (2 mg/kg and 4 mg/kg) showed higher alpha diversity in the rumen microbial community, likely because of the increased VFA concentration during fermentation. This results in a decrease in pH and inhibits acid-labile microbial metabolic activity. Conversely, the high-dose groups (8 mg/kg and 10 mg/kg) had higher total acid content in the rumen fluid than the low-dose 5-HTP groups (2 mg/kg and 4 mg/kg), potentially leading to a decrease in microbial community diversity. The dominant phyla in this study were Bacteroidetes, Firmicutes, Proteobacteria, Actinobacteria, and Verrucomicrobia, with relative abundances ranging from 19.29% to 77.70%, 12.82% to 49.55%, 0.98% to 47.38%, 0.44% to 6.51%, and 0.02% to 3.67%, respectively. These phyla, particularly Verrucomicrobia, are important for maintaining intestinal homeostasis and metabolic activity. Zhang and Wang (36) showed that the two phyla with the highest relative abundances were Bacteroidetes and Firmicutes. Bacteroidetes efficiently decompose carbohydrates in the gastrointestinal tract (37), primarily starch, proteins, and polysaccharides (38), with numerous polysaccharide utilization sites for various enzymes to efficiently degrade polysaccharides (39). Firmicutes are capable of breaking down fibers, including *Ruminococcus*, *Butyrivibrio*, and *Pseudobyrivibrio* species (40). Proteobacteria are Gram-negative bacteria, and *Vibrio succinogenes* belong to the phylum Proteobacteria. The high-dose 5-HTP groups (8 mg/kg and 10 mg/kg) showed a significant increase in the relative abundance of Proteobacteria at 12 and 48 h compared to the low-dose 5-HTP groups (2 mg/kg and 4 mg/kg). This suggests that the high-dose groups (8 mg/kg and 10 mg/kg) were more favorable for the growth and reproduction of Proteobacteria. *Prevotella*, *Succinovibrio*, *Lunella*, and *Succinobacter* were the dominant genera, with relative abundances of 9.55-51.06%, 0.63-45.88%, 0.58-17.64%, and 0.73-5.74%, respectively. The *Prevotella* genus is primarily responsible for the breakdown of xylan and pectin but not cellulose. *Provotella* secretes xylanase and carboxymethyl cellulase. The main components of *Provotella* breakdown products include acetic acid, succinic acid, and propionic acid. *Vibrio succinogenes* breaks down fructan and protein in forage, resulting in the production of fermentation products such as acetic, succinic, formic, and lactic acids. This process promotes protein synthesis (41). This study demonstrated that the high-dose 5-HTP groups (8 mg/kg and 10 mg/kg) showed significant increase in the abundance of *Vibrio succinogenes* at 12 h and 24 h (*P* < 0.05). This finding was consistent with previous research showing a higher production of VFA in the high-dose groups (8 mg/kg and 10 mg/kg). Furthermore, a positive correlation was observed between acetic acid production and *Vibrio succinogenes* in the high-dose 5-HTP groups (8 mg/kg and 10 mg/kg). Beta diversity analysis revealed that the community composition of 5-HTP supplemented at 2 and 4 mg/kg and 8 and 10 mg/kg was similar, indicating that 5-HTP supplementation affected microbial community composition and VFA production.

### 4.3 Effects of 5-HTP on rumen metabolite *in vitro*

In our study, 15 significantly differential metabolites were found, including three alcohols, nine organic acids, one carbohydrate, one nucleoside, and one aldehyde. The results showed that 5-HTP supplementation increased the levels of four metabolites: norvaline, cycloleucine 2, methylmalonic acid, and octanal 2. Additionally, the high-dose groups (8 mg/kg and 10 mg/kg) exhibited down-regulation of uridine 2, lignin acid, and N-methyltryptophan, while seven other metabolites (panthenol 2, lipoic acid, β-sitosterol, gluconic acid 1, N-dimethylarginine, cholesterol, and lyxose 2) showed both down-regulation and up-regulation in low-dose 5-HTP groups (2 mg/kg and 4 mg/kg). The study also analyzed 11 metabolic pathways associated with the differential metabolites, including unsaturated fatty acid biosynthesis and methane metabolism. Overall, 15 differential metabolites were identified as potential chemical markers for future studies on the effects of 5-HTP on ruminal function.

### 4.4 Correlation analysis between rumen metabolome and microorganisms *in vitro*

Pearson’s correlation analysis revealed a positive correlation between 2’-deoxyadenosine and *Prevotella*, 1-hydroxy-2-naphthoic acid and *Succinivibrio*, and 1-mono palmitin, 2’-deoxyadenosine 1, and *Sharpea*. Additionally, ASV Spearman correlation analysis showed that 2-butyne-1,4-diol, 1,3-cyclohexanedione, 1,2,3-dihydropyridine, 1-methylhydantoin, 1,1-hydroxy-2-naphthoic acid, and 2,3-dimethyl succinic acid were positively correlated with *Succinivibrio* and *Selenomonas*. Both Pearson and ASV Spearman correlation analyses indicated a positive correlation between 1-Hydroxy-2-naphthoic acid and *Succinivibrio*, suggesting that the addition of 5-HTP may increase the abundance of this metabolite in the rumen, leading to increased *Succinivibrio* abundance.

### 4.5 Effects of 5-HTP on nutrient digestion and growth performance in sheep

There was no significant difference in DMI between the two groups over 60 days (*P* > 0.05). However, the 5-HTP group gained an average weight of 1.2 kg more than the control group at day 60. The 5-HTP group also showed a significantly higher body weight on day 30 (*P* < 0.05) and had a higher average daily gain than the control group at all growth stages.

### 4.6 Effects of 5-HTP on blood parameters in sheep

Blood biochemical indices in animals indicate their health (42), nutritional levels, and metabolic functions (43). Total protein in plasma, composed of albumin and globulin, transports nutrients, maintains colloid osmotic pressure, and regulates immunity (44). Albumin, synthesized in the liver, protects globulins, stabilizes blood pressure, and enhances immunity and resistance (45). 5-HTP reduced the overall protein and globulin levels in the plasma (*P* < 0.05), possibly due to the increased activity of rumen fibro-degraders and the inhibition of proteolytics. This suggests that 5-HTP stimulation results in decreased protein absorption and metabolism. The 5-HTP group showed a higher albumin concentration on day 15 (*P* < 0.05), indicating a normal body state, but it did not significantly affect the blood antioxidant capacity of sheep (*P* > 0.05). GH levels were also higher in the 5-HTP group, indicating greater weight gain. Additionally, INS concentrations were higher in the 5-HTP group on day 60 (*P* < 0.05), emphasizing the importance of GH in regulating animal growth and metabolism. Similarly, Zeng et al. (18) reported that 5-HTP supplementation in dairy cows increased plasma GH concentrations. Feeding sheep with 5-HTP can stimulate the synthesis of 5-HT, which in turn leads to the secretion of GH in the hypothalamus. This increased secretion of GH results in a higher blood glucose concentration, requiring the 5-HTP group to produce more INS to maintain normal body levels. During the 60-day experimental period, the blood levels of 5-HTP, 5-HT, and MT initially increased and then gradually decreased. The 5-HTP group showed higher levels, indicating a peak in MT synthesis before day 45, followed by a gradual decline.

### 4.7 Effects of 5-HTP on blood metabolite in sheep

Differential metabolites were identified using VIP and *P-*values. A total of 43 compounds were selected based on their FC and *P* values. These metabolites include S-methylmethionine, 5-HTP, Flaulol A, 5-Methoxyvidan, 5-HEPE, cholic acid, nutriacholic acid, Agrocybin, 2-Methylhippuric acid, DL-tyrosine, L-methionine, and phosphatidylcholine. We conducted a pathway enrichment analysis and selected 8 metabolic pathways, including tryptophan metabolism, after bubble map collation. The presence of 5-HTP in the differential metabolites indicates that some fed 5-HTP is not completely metabolized in the rumen and enters the sheep’s bloodstream to be involved in tryptophan metabolic pathways.

## 5 Conclusion

Dietary supplementation of 5-HTP (8 mg/kg DM) improved sheep growth performance by enhancing ruminal functions, antioxidant capacity, and tryptophan metabolism in both *in vitro and in vivo* models. This study shows that 5-HTP has a positive impact on sheep rumen function and growth performance, suggesting its potential as a functional feed additive in ruminants.

## Data availability statement

The NCBI Sequence Read Archive (SRA) database contains the raw sequence reads for all samples described in the study (No.PRJNA1014763).

## Ethics statement

The animal study was approved by the Institutional Animal Care Committee of Jilin Agricultural University (JLAUACUC2022-003) for the management of experimental animals. The studies were conducted in accordance with the local legislation and institutional requirements. Written informed consent was obtained from the owners for the participation of their animals in this study. The study was conducted in accordance with the local legislation and institutional requirements.

## Author contributions

ZS: Conceptualization, Supervision, Writing – review & editing. NA: Methodology, Data curation, Software, Writing – original draft. LC: Methodology, Formal analysis, Software, Writing – original draft. YX: Methodology, Writing – original draft. LZ: Methodology, Writing – original draft. GY: Methodology, Writing – original draft. SW: Methodology, Writing – original draft. ZW: Writing – original draft. JD: Methodology, Writing – original draft. WZhan: Writing – original draft. WZhao: Methodology, Writing – original draft. GQ: Conceptualization, Supervision, Writing – review & editing. XZ: Conceptualization, Supervision, Writing – review & editing. RZ: Conceptualization, Writing – review & editing. YZ: Conceptualization, Supervision, Writing – review & editing. TW: Conceptualization, Supervision, Writing – review & editing.
