# Supplementary material for: Dietary 5-hydroxytryptophan improves sheep growth performance by enhancing ruminal functions, antioxidant capacity, and tryptophan metabolism: in vitro and in vivo studies
Source: Front Immunol. 2024 May 21;15:1398310. doi: 10.3389/fimmu.2024.1398310 (PMC11148369; doi:10.3389/fimmu.2024.1398310)
Supplement: Supplementary file 1 [file Table_1.docx]

Supplementary TABLE S1. Ingredients and chemical composition of the experimental diet (% of DM).

| Ingredients | | Chemical Composition (% DM) | |
| --- | --- | --- | --- |
| Corn | 35.00 | Dry matter | 90.20 |
| Soybean meal | 13.00 | Crude protein | 14.27 |
| Corn germ meal | 6.00 | Neutral detergent fiber | 28.17 |
| Corn bran | 10.00 | Acid detergent fiber | 18.35 |
| Peanut hull | 23.00 | Starch | 25.87 |
| [Bentonite](javascript:;) | 4.00 | Ash | 11.30 |
| Sucrose | 4.00 | Ether extract | 3.94 |
| Premix^1^ | 5.00 | Calcium | 0.61 |
| Total | 100.00 |  |  |

^1^Nutrient content of premix (per kg): Ca^2+^ = 110 mg, Cu^2+^= 140 mg, Zn^2+^= 930 mg, Mn^2+^= 600 mg, Co^2+^ =13 mg, I^-^ = 20 mg, Se^4+^= 13 mg, Vitamin A = 340 KIU, Vitamin D3 = 120 KIU, Vitamin E = 1700 IU.

Supplementary TABLE S2. Effects of 5-HTP supplementation on dominant bacterial phylum and genus composition at different fermentation times (%) *in vitro*.

| Item | Time | Control | 5-HTP doses (mg/kg DM) | | | | SEM | *P*-value | | |
| --- | --- | --- | --- | --- | --- | --- | --- | --- | --- | --- |
|  |  | 0 | 2 | 4 | 8 | 10 |  | Dose | Time | D×T |
| Phylum |  |  | | | | |  |  |  |  |
| Bacteroidetes | 0h | 57.29 | 57.27 | 57.28 | 57.30 | 57.26 | 1.70 | <0.01 | <0.01 | <0.01 |
|  | 12h | 67.47^a^ | 76.02^a^ | 77.70^a^ | 51.21^b^ | 47.67^b^ | 3.24 |  |  |  |
|  | 48h | 23.62^ab^ | 30.67^a^ | 27.91^ab^ | 20.14^b^ | 19.29^b^ | 1.46 |  |  |  |
| Firmicutes | 0h | 33.45 | 33.46 | 33.46 | 33.45 | 33.44 | 1.55 | <0.01 | <0.01 | <0.05 |
|  | 12h | 12.82 | 17.28 | 15.25 | 13.52 | 13.87 | 0.77 |  |  |  |
|  | 48h | 39.76^a^ | 44.41^a^ | 49.55^a^ | 38.26^ab^ | 27.55^b^ | 2.27 |  |  |  |
| Proteobacteria | 0h | 2.89 | 2.88 | 2.88 | 2.89 | 2.89 | 0.21 | <0.01 | <0.01 | 0.64 |
|  | 12h | 15.38^b^ | 0.98^c^ | 1.56^c^ | 32.16^a^ | 35.49^a^ | 3.70 |  |  |  |
|  | 48h | 27.08^bc^ | 13.02^cd^ | 7.87^d^ | 32.52^ab^ | 47.38^a^ | 3.90 |  |  |  |
| Actinobacteria | 0h | 2.88 | 2.87 | 2.85 | 2.88 | 2.88 | 0.28 | <0.01 | 0.35 | 0.25 |
|  | 12h | 0.56 | 0.83 | 0.44 | 0.60 | 0.69 | 0.10 |  |  |  |
|  | 48h | 3.93 | 4.10 | 6.51 | 6.27 | 2.94 | 0.65 |  |  |  |
| Verrucomicrobia | 0h | 0.02 | 0.02 | 0.02 | 0.02 | 0.02 | 0.00 | <0.01 | <0.01 | <0.05 |
|  | 12h | 0.96^ab^ | 1.49^a^ | 1.46^a^ | 0.66^b^ | 0.68^b^ | 0.13 |  |  |  |
|  | 48h | 2.07^ab^ | 3.67^a^ | 3.65^a^ | 0.57^b^ | 0.88^b^ | 0.37 |  |  |  |
| Genus | | | | | | | | | | |
| *Prevotella* | 0h | 44.75 | 44.65 | 44.72 | 44.75 | 44.75 | 1.53 | <0.01 | <0.01 | <0.05 |
|  | 12h | 46.77^a^ | 50.33^a^ | 51.06^a^ | 37.03^b^ | 34.26^b^ | 1.94 |  |  |  |
|  | 48h | 15.24^a^ | 17.27^a^ | 15.02^a^ | 9.55^b^ | 12.46^ab^ | 0.87 |  |  |  |
| *Succinivibrio* | 0h | 2.69 | 2.69 | 2.69 | 2.69 | 2.69 | 0.19 | <0.01 | <0.01 | 0.65 |
|  | 12h | 15.13^b^ | 0.63^c^ | 1.18^c^ | 31.72^a^ | 35.14^a^ | 3.69 |  |  |  |
|  | 48h | 24.17^b^ | 2.66^c^ | 2.76^c^ | 30.92^ab^ | 45.88^a^ | 4.41 |  |  |  |
| *Selenomonas* | 0h | 1.07 | 1.07 | 1.06 | 1.07 | 1.08 | 0.05 | <0.01 | 0.47 | 0.29 |
|  | 12h | 0.58^b^ | 0.70^b^ | 0.80^b^ | 0.86^b^ | 1.48^a^ | 0.10 |  |  |  |
|  | 48h | 16.73 | 14.78 | 17.64 | 12.30 | 9.88 | 1.43 |  |  |  |
| *Succiniclasticum* | 0h | 2.06 | 2.08 | 2.07 | 2.07 | 2.07 | 0.13 | <0.01 | <0.01 | <0.01 |
|  | 12h | 1.03^b^ | 1.77^a^ | 1.54^a^ | 0.83^b^ | 0.76^b^ | 0.10 |  |  |  |
|  | 48h | 3.76^ab^ | 5.74^a^ | 5.22^a^ | 1.22^c^ | 2.13^bc^ | 0.50 |  |  |  |
| *Sharpea* | 0h | 5.76 | 5.75 | 5.74 | 5.76 | 5.74 | 0.83 | <0.01 | <0.01 | <0.01 |
|  | 12h | 0.97 | 0.82 | 0.77 | 2.00 | 1.55 | 0.22 |  |  |  |
|  | 48h | 0.88^b^ | 0.43^b^ | 0.54^b^ | 5.56^a^ | 1.84^b^ | 0.51 |  |  |  |

^a,b^ Different superscripts in the same raw implies their mean values are significantly different (*P* ≤ 0.05).

Supplementary TABLE S3. Differential metabolites information under 5-HTP supplementation at different fermentation times *in vitro*.

| Metabolites | VIP | *P-*value | FC |
| --- | --- | --- | --- |
| A12.0T vs A12.2T (7) | | | |
| 4-Hydroxyphenylacetic acid | 2.45 | 0.029 | 0.62 |
| Lignoceric acid | 2.51 | 0.038 | 1.49 |
| 3-Hydroxybutyric acid | 2.03 | 0.038 | 0.78 |
| Hexadecane | 2.89 | 0.008 | 1.56 |
| D-Talose 1 | 1.78 | 0.029 | 0.58 |
| 4',7-Dihyroxyflavanone 1 | 2.04 | 0.041 | 1.32 |
| 2-Deoxyuridine | 2.41 | 0.011 | 0.75 |
| A12.0T vs A12.4T (32) | | | |
| Octanal 2 | 2.41 | 0.000 | 0.34 |
| Cholesterol-2,2,3,4,4,6-d6 | 2.04 | 0.004 | 3.08 |
| Allylmalonic acid | 1.89 | 0.020 | 1.21 |
| 3-Hydroxypyruvate | 2.38 | 0.000 | 0.38 |
| 4-Hydroxyphenylacetic acid | 1.93 | 0.019 | 0.60 |
| Methylmalonic acid | 2.45 | 0.000 | 0.33 |
| N,N-dimethylarginine | 2.38 | 0.000 | 2.14 |
| Palmitic acid | 1.75 | 0.040 | 1.43 |
| Cytidine-monophosphate 1 | 1.87 | 0.012 | 2.02 |
| 2-Keto-isovaleric acid 1 | 1.97 | 0.002 | 0.74 |
| Thioctamide 1 | 1.96 | 0.004 | 1.62 |
| Hexadecane | 2.15 | 0.005 | 1.97 |
| Panthenol 2 | 2.10 | 0.001 | 2.11 |
| 3-Hexenedioic acid | 1.87 | 0.010 | 1.49 |
| 2-Ketocaproic acid | 1.89 | 0.015 | 0.61 |
| N-Acetyl-L-leucine 1 | 2.34 | 0.000 | 1.58 |
| N-cyclohexylformamide 1 | 1.76 | 0.028 | 1.18 |
| Glycocyamine 1 | 2.12 | 0.002 | 1.55 |
| N-Methyl-L-glutamic acid 2 | 2.36 | 0.000 | 1.76 |
| 2-Methylfumarate | 1.93 | 0.016 | 1.45 |
| Beta-Sitosterol | 2.00 | 0.005 | 4.54 |
| 1,3-Cyclohexanedione 1 | 2.00 | 0.008 | 1.25 |
| Dioctyl phthalate | 1.89 | 0.010 | 1.22 |
| Hexachlorobenzene | 1.50 | 0.039 | 1.36 |
| Norvaline | 2.41 | 0.000 | 0.23 |
| 1-Methylhydantoin 1 | 1.90 | 0.015 | 1.23 |
| Gluconic acid 1 | 1.89 | 0.007 | 2.16 |
| Lipoic acid | 2.32 | 0.000 | 2.01 |
| Lactic acid | 1.70 | 0.041 | 0.66 |
| Cycloleucine 2 | 2.43 | 0.000 | 0.16 |
| D-erythronolactone 2 | 2.26 | 0.001 | 1.87 |
| Glutamine 3 | 2.23 | 0.000 | 1.41 |
| A12.0T vs A12.8T (29) | | | |
| Octanal 2 | 2.26 | 0.002 | 0.55 |
| 2'-Deoxyguanosine 1 | 2.17 | 0.008 | 1.97 |
| Squalene | 2.34 | 0.002 | 1.73 |
| 3-Hydroxypyruvate | 2.23 | 0.003 | 0.60 |
| Methylmalonic acid | 2.42 | 0.000 | 0.50 |
| N,N-dimethylarginine | 2.04 | 0.016 | 1.41 |
| Palmitic acid | 2.09 | 0.008 | 1.34 |
| Uridine 2 | 2.35 | 0.005 | 2.22 |
| 7,8-Dimethylalloxazine | 1.71 | 0.049 | 1.32 |
| Lignoceric acid | 2.31 | 0.006 | 2.26 |
| Behenic acid | 2.24 | 0.011 | 2.31 |
| Thioctamide 1 | 1.64 | 0.049 | 1.42 |
| 6-Phosphogluconic acid | 2.16 | 0.009 | 1.52 |
| Panthenol 2 | 1.79 | 0.024 | 1.56 |
| 2-Ketocaproic acid | 1.68 | 0.038 | 0.68 |
| Melatonin 2 | 1.72 | 0.048 | 1.67 |
| N-Acetyl-L-leucine 1 | 1.77 | 0.034 | 1.22 |
| Octadecanol | 2.40 | 0.002 | 1.59 |
| Cis-gondoic acid | 1.86 | 0.031 | 1.52 |
| 1-Aminocyclopropanecarboxylic acid | 1.99 | 0.011 | 1.23 |
| 2-Methylfumarate | 1.72 | 0.035 | 1.26 |
| Norvaline | 2.51 | 0.001 | 0.36 |
| N-methyltryptophan | 2.07 | 0.009 | 2.22 |
| Lipoic acid | 1.86 | 0.021 | 1.44 |
| Heptadecanoic acid | 2.34 | 0.001 | 1.75 |
| Biotin | 1.76 | 0.041 | 1.48 |
| Cycloleucine 2 | 2.56 | 0.000 | 0.27 |
| D-erythronolactone 2 | 1.85 | 0.029 | 1.34 |
| 2-Ketovaleric acid 2 | 1.72 | 0.039 | 1.33 |
| A12.0T vs A12.10T (27) | | | |
| Octanal 2 | 2.40 | 0.001 | 0.47 |
| Glutaraldehyde 1 | 1.93 | 0.012 | 0.85 |
| Maleamate 3 | 1.81 | 0.019 | 0.74 |
| Malonamide 3 | 1.66 | 0.035 | 0.75 |
| Canavanine degr prod | 2.20 | 0.002 | 0.77 |
| 3,4-Dihydroxypyridine | 1.64 | 0.037 | 0.70 |
| Piceatannol 1 | 1.87 | 0.018 | 0.51 |
| 3-Hydroxypyruvate | 2.35 | 0.002 | 0.54 |
| 4-Hydroxyphenylacetic acid | 1.60 | 0.036 | 0.55 |
| 4-Methylcatechol | 1.78 | 0.020 | 0.74 |
| Methylmalonic acid | 2.51 | 0.000 | 0.42 |
| N,N-dimethylarginine | 1.90 | 0.015 | 1.25 |
| Xanthurenic acid | 1.75 | 0.036 | 2.35 |
| 2-Keto-isovaleric acid 1 | 1.96 | 0.011 | 0.82 |
| Oxalic acid | 1.96 | 0.009 | 0.80 |
| Succinate semialdehyde 2 | 1.92 | 0.010 | 0.78 |
| 2,3-Dihydroxypyridine | 1.70 | 0.038 | 0.87 |
| Alpha-ketoisocaproic acid 2 | 1.93 | 0.011 | 0.58 |
| Dihydroxyacetone | 2.38 | 0.002 | 0.80 |
| Norvaline | 2.45 | 0.001 | 0.36 |
| N-Carbamylglutamate 4 | 1.70 | 0.036 | 1.44 |
| Carbobenzyloxy-L-leucine degr1 | 1.59 | 0.046 | 0.82 |
| Cycloleucine 2 | 2.28 | 0.001 | 0.36 |
| 3-(3-Hydroxyphenyl) propionic acid | 1.65 | 0.047 | 0.25 |
| 3-Aminoisobutyric acid 1 | 1.61 | 0.046 | 0.75 |
| 2-Amino-2-methylpropane-1,3-diol 2 | 2.29 | 0.001 | 0.77 |
| 2-Ketovaleric acid 2 | 1.65 | 0.036 | 0.65 |
| A48.0T vs A48.2T (7) | | | |
| Galactonic acid | 2.47 | 0.010 | 0.35 |
| O-acetylserine 1 | 2.41 | 0.021 | 0.72 |
| Beta-Alanine 2 | 2.46 | 0.007 | 0.63 |
| Lyxose 2 | 2.55 | 0.003 | 0.48 |
| 4-Aminobutyric acid 1 | 2.44 | 0.014 | 0.53 |
| 5-Aminovaleric acid 1 | 2.31 | 0.021 | 0.41 |
| 4-Hydroxyphenylethanol | 1.83 | 0.042 | 0.75 |
| A48.0T vs A48.4T (33) | | | |
| Octanal 2 | 1.97 | 0.023 | 0.32 |
| Cholesterol-2,2,3,4,4,6-d6 | 2.25 | 0.000 | 2.23 |
| Allylmalonic acid | 1.55 | 0.035 | 1.35 |
| 3-Hydroxypyruvate | 2.23 | 0.004 | 0.37 |
| Methylmalonic acid | 2.59 | 0.010 | 0.25 |
| N,N-dimethylarginine | 2.09 | 0.002 | 1.88 |
| 2-Hydroxy-3-isopropylbutanedioic acid | 1.81 | 0.029 | 0.67 |
| 2-Butyne-1,4-diol | 1.55 | 0.035 | 1.38 |
| O-Acetylserine 1 | 1.99 | 0.003 | 0.72 |
| Citraconic acid degr1 | 1.56 | 0.033 | 1.38 |
| Cytidine-monophosphate 1 | 1.64 | 0.029 | 1.61 |
| Panthenol 2 | 1.97 | 0.011 | 1.70 |
| 3-Hexenedioic acid | 1.93 | 0.009 | 1.73 |
| 2-Ketocaproic acid | 2.34 | 0.001 | 0.54 |
| 2,3-Dihydroxypyridine | 1.63 | 0.026 | 1.41 |
| N-Acetyl-L-leucine 1 | 1.89 | 0.007 | 1.57 |
| Maleimide | 1.49 | 0.044 | 1.35 |
| N-cyclohexylformamide 1 | 1.48 | 0.044 | 1.34 |
| Glycocyamine 1 | 1.91 | 0.009 | 1.51 |
| N-Methyl-L-glutamic acid 2 | 1.73 | 0.014 | 1.52 |
| Trans,trans-Muconic acid | 1.88 | 0.013 | 0.75 |
| Beta-Sitosterol | 1.66 | 0.024 | 2.28 |
| 1,3-Cyclohexanedione 1 | 1.56 | 0.035 | 1.35 |
| Norvaline | 2.59 | 0.000 | 0.18 |
| 1-Methylhydantoin 1 | 1.56 | 0.033 | 1.37 |
| Cytosin | 1.58 | 0.043 | 0.82 |
| Gluconic acid 1 | 1.49 | 0.040 | 1.29 |
| Biuret 3 | 1.53 | 0.035 | 1.33 |
| Lipoic acid | 2.09 | 0.003 | 1.71 |
| Cycloleucine 2 | 2.61 | 0.000 | 0.12 |
| D-erythronolactone 2 | 2.22 | 0.001 | 1.93 |
| Glutamine 3 | 1.56 | 0.048 | 1.32 |
| 2-Ketovaleric acid 2 | 1.71 | 0.048 | 1.81 |
| A48.0T vs A48.8T (27) | | | |
| Octanal 2 | 1.92 | 0.027 | 0.34 |
| Cholesterol-2,2,3,4,4,6-d6 | 1.93 | 0.017 | 2.10 |
| 2'-Deoxyguanosine 1 | 1.95 | 0.011 | 1.67 |
| 3-Hydroxypyruvate | 1.74 | 0.029 | 0.54 |
| Methylmalonic acid | 2.59 | 0.010 | 0.27 |
| Uridine 2 | 1.98 | 0.008 | 1.52 |
| 2-Hydroxy-3-isopropylbutanedioic acid | 1.89 | 0.040 | 0.70 |
| Lignoceric acid | 1.73 | 0.027 | 1.76 |
| Behenic acid | 1.62 | 0.039 | 1.64 |
| O-acetylserine 1 | 1.70 | 0.018 | 0.82 |
| Cytidine-monophosphate 1 | 1.74 | 0.036 | 1.76 |
| Thioctamide 1 | 1.63 | 0.048 | 1.55 |
| (S)-Mandelic acid | 1.96 | 0.011 | 1.35 |
| 6-phosphogluconic acid | 1.50 | 0.045 | 1.87 |
| Hexadecane | 1.62 | 0.049 | 0.63 |
| Panthenol 2 | 1.84 | 0.031 | 1.71 |
| 3-Hexenedioic acid | 1.74 | 0.042 | 1.57 |
| 2-Ketocaproic acid | 2.22 | 0.002 | 0.61 |
| Guanosine-5'-monophosphate | 1.98 | 0.021 | 1.74 |
| Trans,trans-Muconic acid | 1.64 | 0.046 | 0.81 |
| Hexachlorobenzene | 1.85 | 0.028 | 1.31 |
| Norvaline | 2.60 | 0.000 | 0.20 |
| Lipoic acid | 1.79 | 0.038 | 1.66 |
| Cycloleucine 2 | 2.52 | 0.000 | 0.21 |
| D-erythronolactone 2 | 1.87 | 0.029 | 1.81 |
| Cerotinic acid | 1.62 | 0.034 | 2.37 |
| 2-Ketovaleric acid 2 | 1.83 | 0.042 | 2.27 |
| A48.0T vs A48.10T (27) | | | |
| Octanal 2 | 2.09 | 0.019 | 0.27 |
| Cholesterol-2,2,3,4,4,6-d6 | 2.03 | 0.011 | 2.09 |
| 2-Ketobutyric acid 2 | 1.94 | 0.027 | 3.91 |
| 3-Hydroxypyruvate | 2.14 | 0.012 | 0.50 |
| Methylmalonic acid | 2.66 | 0.010 | 0.26 |
| N,N-dimethylarginine | 2.10 | 0.007 | 1.73 |
| 2-Hydroxy-3-isopropylbutanedioic acid | 1.98 | 0.025 | 0.66 |
| Succinic acid | 1.78 | 0.047 | 0.53 |
| Maltose | 1.77 | 0.039 | 0.48 |
| Cyclohexane-1,2-diol | 1.85 | 0.027 | 1.28 |
| O-Phosphorylethanolamine | 2.00 | 0.012 | 1.89 |
| Thioctamide 1 | 1.82 | 0.023 | 1.47 |
| (S)-Mandelic acid | 1.79 | 0.025 | 1.30 |
| 3-Hexenedioic acid | 1.95 | 0.017 | 1.58 |
| Inosine 5'-monophosphate | 1.76 | 0.033 | 1.42 |
| Trehalose | 1.95 | 0.028 | 0.19 |
| 2-Ketocaproic acid | 2.12 | 0.006 | 0.67 |
| 3,6-Anhydro-D-galactose 3 | 2.02 | 0.010 | 1.64 |
| N-Acetyl-L-leucine 1 | 1.84 | 0.024 | 1.47 |
| Glycocyamine 1 | 1.95 | 0.018 | 1.49 |
| N-Methyl-L-glutamic acid 2 | 1.65 | 0.049 | 1.43 |
| Hexachlorobenzene | 2.05 | 0.008 | 1.44 |
| Norvaline | 2.65 | 0.000 | 0.17 |
| Lipoic acid | 2.04 | 0.012 | 1.66 |
| 2-Amino-3-methoxybenzoic acid 1 | 1.66 | 0.050 | 0.80 |
| Cycloleucine 2 | 2.21 | 0.004 | 0.40 |
| D-erythronolactone 2 | 2.16 | 0.006 | 1.80 |

Where: VIP- variable influence on projection; FC- fold change values. A12.0T, A12.2T, A12.4T, A12.8T and A12.10T imply fermentation for 12 h, and 0, 2, 4, 8 and 10 mg/kg 5-HTP supplementation, respectively. A48.0T, A48.2T, A48.4T, A48.8T, and A48.10T indicated fermentation for 48 h, and 0, 2, 4, 8 and 10 mg/kg 5-HTP supplementation, respectively. A total of 10 sets of experiments, each group of 6 parallel samples, the total number of samples n = 60.

Supplementary TABLE S4. Comparison of metabolites and pathways under 5-HTP supplementation at different fermentation times *in vitro*.

| Pathway | Metabolite | Group |
| --- | --- | --- |
| ABC transporters  Alanine, aspartate and glutamate metabolism  Aminobenzoate degradation  Antifolate resistance  Bacterial chemotaxis  Benzoate degradation  Biosynthesis of alkaloids derived from histidine and purine  Biosynthesis of alkaloids derived from ornithine, lysine and nicotinic acid  Biosynthesis of alkaloids derived from shikimate pathway  Biosynthesis of alkaloids derived from terpenoid and polyketide  Biosynthesis of alkaloids derived from terpenoid and polyketide  Biosynthesis of cofactors  Biosynthesis of phenylpropanoids  Biosynthesis of plant hormones  Biosynthesis of plant secondary metabolites  Biosynthesis of secondary metabolites  Biosynthesis of terpenoids and steroids  Biosynthesis of unsaturated fatty acids  Biosynthesis of various alkaloids  Biosynthesis of various plant secondary metabolites  Biotin metabolism  Bisphenol degradation  Butanoate metabolism  Butanoate metabolism  C5-Branched dibasic acid metabolism  cAMP signaling pathway  cAMP signaling pathway  Carbohydrate digestion and absorption  Carbon fixation pathways in prokaryotes  Carbon metabolism  Cationic antimicrobial peptide (CAMP) resistance  Central carbon metabolism in cancer  cGMP-PKG signaling pathway  Chemical carcinogenesis - receptor activation  Chloroalkane and chloroalkene degradation  Chlorocyclohexane and chlorobenzene degradation  Citrate cycle （TCA cycle）  Cutin, suberine and wax biosynthesis  Cysteine and methionine metabolism  Degradation of aromatic compounds  Fatty acid biosynthesis  Fatty acid degradation  Fatty acid elongation  Fatty acid metabolism  GABAergic synapse  Galactose metabolism  Glucagon signaling pathway  Glycerolipid metabolism  Glycerophospholipid metabolism  Glycine, serine and threonine metabolism  Glycosylphosphatidylinositol (GPI)-anchor biosynthesis  Glyoxylate and dicarboxylate metabolism  Lipoic acid metabolism  Lysine degradation  Metabolic pathways  Methane metabolism  Microbial metabolism in diverse environments  Naphthalene degradation  Nicotinate and nicotinamide metabolism  Nucleotide metabolism  Olfactory transduction  Oxidative phosphorylation  Pentose phosphate pathway  Phenylalanine metabolism  Phosphotransferase system (PTS)  Phototransduction  Propanoate metabolism  Purine metabolism  Pyrimidine metabolism  Pyruvate metabolism  Riboflavin metabolism  Sesquiterpenoid and triterpenoid biosynthesis  Sphingolipid metabolism  Sphingolipid signaling pathway  Starch and sucrose metabolism  Steroid biosynthesis  Sulfur metabolism  Taste transduction  Toluene degradation  Tryptophan metabolism  Two-component system  Tyrosine metabolism  Tyrosine metabolism  Valine, leucine and isoleucine degradation  Vancomycin resistance  Vitamin digestion and absorption  Xylene degradation | (R)-Lipoic acid | A12.0T_vs_A12.4T A12.0T_vs_A12.8T A48.0T_vs_A48.10T A48.0T_vs_A48.4T A48.0T_vs_A48.8T |
|  |  |  |
|  |  |  |
|  |  |  |
|  |  |  |
|  | (S)-3-Hydroxybutyric acid | A12.0T_vs_A12.2T |
|  | 1-Aminocyclopropanecarboxylic acid | A12.0T_vs_A12.8T |
|  | 4-Hydroxyphenyl acetate | A12.0T_vs_A12.10T A12.0T_vs_A12.2T A12.0T_vs_A12.4T |
|  |  |  |
|  |  |  |
|  | 4-Methylcatechol | A12.0T_vs_A12.10T |
|  | D-Lactic acid | A12.0T_vs_A12.4T |
|  | D-Maltose | A48.0T_vs_A48.10T |
|  | Galactonic acid | A48.0T_vs_A48.2T |
|  | Guanosine monophosphate | A48.0T_vs_A48.8T |
|  | Hydroxypyruvic acid | A12.0T_vs_A12.10T A12.0T_vs_A12.4T A12.0T_vs_A12.8T A48.0T_vs_A48.10T A48.0T_vs_A48.4T A48.0T_vs_A48.8T |
|  |  |  |
|  |  |  |
|  |  |  |
|  |  |  |
|  |  |  |
|  | Inosinic acid | A48.0T_vs_A48.10T |
|  | Hexachlorobenzene | A12.0T_vs_A12.4T A48.0T_vs_A48.8T A48.0T_vs_A48.10T |
|  |  |  |
|  |  |  |
|  | 5-Hydroxyphenyl acetate | A12.0T_vs_A12.4T A12.0T_vs_A12.10T |
|  |  |  |
|  | 6-Phosphogluconic acid | A12.0T_vs_A12.8T A48.0T_vs_A48.8T |
|  |  |  |
|  | Behenate | A12.0T_vs_A12.8T A48.0T_vs_A48.8T |
|  |  |  |
|  | Beta-Sitosterol | A12.0T_vs_A12.4T A48.0T_vs_A48.4T |
|  |  |  |
|  | Biotin | A12.0T_vs_A12.8T |
|  | Cytosine | A48.0T_vs_A48.4T |
|  | Deoxyuridine | A12.0T_vs_A12.2T |
|  | Dihydroxyacetone | A12.0T_vs_A12.10T |
|  | Dioctyl phthalate | A12.0T_vs_A12.4T |
|  | Lumichrome | A12.0T_vs_A12.8T |
|  | Mesaconic acid | A12.0T_vs_A12.4T A12.0T_vs_A12.8T |
|  |  |  |
|  | Mandelic acid | A48.0T_vs_A48.8T A48.0T_vs_A48.10T |
|  |  |  |
|  | Methylmalonic acid | A12.0T_vs_A12.10T A12.0T_vs_A12.4T A12.0T_vs_A12.8T A48.0T_vs_A48.10T A48.0T_vs_A48.4T A48.0T_vs_A48.8T |
|  |  |  |
|  |  |  |
|  |  |  |
|  |  |  |
|  |  |  |
|  | Methylmalonic beta-Sitosterol | A12.0T_vs_A12.4T |
|  | O-Phosphoethanolamine | A48.0T_vs_A48.10T |
|  | Oxalic acid | A12.0T_vs_A12.10T |
|  | Palmitic acid | A12.0T_vs_A12.4T A12.0T_vs_A12.8T |
|  |  |  |
|  | Palmitic acid;Squalene | A12.0T_vs_A12.8T |
|  | Succinic acid | A48.0T_vs_A48.10T |
|  | Tetracosanoic acid | A12.0T_vs_A12.2T A48.0T_vs_A48.8T A12.0T_vs_A12.8T |
|  |  |  |
|  |  |  |
|  | Trans-trans-Muconic acid | A48.0T_vs_A48.4T A48.0T_vs_A48.8T |
|  |  |  |
|  | Trehalose | A48.0T_vs_A48.10T |
|  | Tyrosol | A48.0T_vs_A48.2T |
|  | Xanthurenic acid | A12.0T_vs_A12.10T |

Where: A12.0T, A12.2T, A12.4T, A12.8T and A12.10T imply fermentation for 12 h, and 0, 2, 4, 8 and 10 mg/kg 5-HTP supplementation respectively; A48.0T, A48.2T, A48.4T, A48.8T, and A48.10T indicated fermentation for 48 h, and 0, 2, 4, 8 and 10 mg/kg 5-HTP supplementation, respectively. A total of 10 sets of experiments, each group of 6 parallel samples, the total number of samples n=60.
